# Differentially expressed *myo*-inositol monophosphatase gene (*CaIMP*) in chickpea (*Cicer arietinum* L.) encodes a lithium-sensitive phosphatase enzyme with broad substrate specificity and improves seed germination and seedling growth under abiotic stresses

**DOI:** 10.1093/jxb/ert336

**Published:** 2013-10-11

**Authors:** Saurabh C. Saxena, Prafull Salvi, Harmeet Kaur, Pooja Verma, Bhanu Prakash Petla, Venkateswara Rao, Nitin Kamble, Manoj Majee

**Affiliations:** National Institute of Plant Genome Research (NIPGR), Aruna Asaf Ali Marg, New Delhi 110067, India

**Keywords:** Ascorbate, gluconeogenesis, inositol, multifunctional, phosphatase, seed germination, stress tolerance.

## Abstract

*myo*-Inositol monophosphatase (IMP) is an essential enzyme in the *myo*-inositol metabolic pathway where it primarily dephosphorylates *myo*-inositol 1-phosphate to maintain the cellular inositol pool which is important for many metabolic and signalling pathways in plants. The stress-induced increased accumulation of inositol has been reported in a few plants including chickpea; however, the role and regulation of IMP is not well defined in response to stress. In this work, it has been shown that IMP activity is distributed in all organs in chickpea and was noticeably enhanced during environmental stresses. Subsequently, using degenerate oligonucleotides and RACE strategy, a full-length IMP cDNA (*CaIMP*) was cloned and sequenced. Biochemical study revealed that *CaIMP* encodes a lithium-sensitive phosphatase enzyme with broad substrate specificity, although maximum activity was observed with the *myo*-inositol 1-phosphate and l-galactose 1-phosphate substrates. Transcript analysis revealed that *CaIMP* is differentially expressed and regulated in different organs, stresses and phytohormones. Complementation analysis in *Arabidopsis* further confirmed the role of CaIMP in l-galactose 1-phosphate and *myo*-inositol 1-phosphate hydrolysis and its participation in *myo*-inositol and ascorbate biosynthesis. Moreover, *Arabidopsis* transgenic plants over-expressing *CaIMP* exhibited improved tolerance to stress during seed germination and seedling growth, while the VTC4/IMP loss-of-function mutants exhibited sensitivity to stress. Collectively, CaIMP links various metabolic pathways and plays an important role in improving seed germination and seedling growth, particularly under stressful environments.

## Introduction

*myo*-Inositol is a six carbon cyclohexane hexitol and has a diverse role in plant biology. Besides a requirement in plant growth and development, *myo-*inositol is also used as a precursor and substrate for many crucial metabolites in plants such as phytate, phosphatidylinositol, galactinol, ascorbate, indole acetic acid conjugate, ononitol, and pinitol. These inositol derivatives were shown to be implicated in various physiological processes including plant stress adaptation (Gross and Boss, 1993; Loewus and Murthy, 2000; Downes *et al.*, 2005; Nunes *et al.*, 2006; Meng *et al.*, 2009; Chaouch and Noctor, 2010; Donahue *et al.*, 2010). The *de novo* synthesis of *myo-*inositol is a two-step process and is highly conserved among biological organisms. In the first step, d-glucose 6-phosphate is converted to l- *myo-*inositol 1-phosphate by the rate-limiting enzyme l-*myo*-inositol 1-phosphate synthase (MIPS; EC 5.5.1.4). In the second step, l-*myo*-inositol 1-phosphate is dephosphorylated to produce free *myo*-inositol by a specific Mg^2+^-dependent *myo*-inositol 1-phosphate phosphatase (IMP; EC 3.1.3.25) (Loewus and Loewus, 1983; Loewus,1990). Beside this, IMP is also reported to dephosphorylate other inositol phosphate compounds such as the breakdown products of phosphoinositides (Gillaspy *et al.*, 1995; Quintero *et al.*, 1996; Loewus and Murthy, 2000). Thus, this enzyme is essential because IMP is required for both *de novo* synthesis as well as the recycling of inositol and can be a critical and potential regulatory point for all pathways that use free inositol.

Even though molecular, biochemical, and functional studies on the *MIPS* gene and enzyme have been studied in great detail in a wide range of organisms, such studies are very much limited in the case of IMP (Majee *et al.*, 2004; Meng *et al.*, 2009; Chaouch and Noctor, 2010; Donahue *et al.*, 2010).

The first *IMP* coding sequence was reported from bovine brain tissue (Diehl *et al.*, 1990). Subsequently, the *IMP* gene has been reported from several prokaryotic and eukaryotic sources. In the case of higher plants, IMP protein is encoded by one (*Arabidopsis*, barley, rice) or multiple (tomato) genes (Suzuki *et al.*, 2007; Fu *et al.*, 2008; Torabinejad *et al.*, 2009). In tomato, IMP enzymes are encoded by a family of three distinct genes which are differentially expressed and developmentally regulated (Gillaspy *et al.*, 1995; Styer *et al.*, 2004). In addition to *IMP*, *IMP*-like genes (*IMPL*) have also been reported in plants. In the case of *Arabidopsis*, two *IMP* -like genes, *IMPL1* and *IMPL2*, have been reported and *IMPL2* has been shown to be involved in histidine biosynthesis (Torabinejad *et al.*, 2009; Petersen *et al.*, 2010).

The IMP proteins belong to a superfamily of metal-dependent phosphatases with broad substrate specificity (Neuwald *et al.*, 1992; Atack *et al.*, 1995). Studies have also revealed the bi-functional activity of IMP protein from mammals (bovine, human, rat) and *Methanococcus* (Parthasarathy *et al.*, 1997; Stec *et al.*, 2000). Similarly, the IMP enzyme from *Arabidopsis* and kiwifruit (*Actinidia deliciosa*) was also shown to catalyse both *myo*-inositol 1-phosphate and l-galactose 1-phosphate dephosphorylation and was considered to be a bi-functional enzyme which participates in both inositol and ascorbate biosynthesis (Laing *et al.*, 2004; Torabinejad *et al.*, 2009). The physiological significance of the bi-functionality of this enzyme is, however, not well understood. Interestingly, soybean IMP has multiple substrate specificity which includes substrates such as sodium pyrophosphate (NaPPi), phytate, and *myo*-inositol 1-phosphate (Islas-Flores and Villanueva, 2007).

Inositol metabolism was previously shown to play an important role in chickpea, particularly in response to drought stress. The stress-induced increased accumulation of *myo*-inositol as a consequence of the transcriptional induction of MIPS has also been reported in chickpea (Boominathan *et al.*, 2004). In our previous study, it was observed that the MIPS enzyme is encoded by two divergent genes, *CaMIPS1* and *CaMIPS2*, and *CaMIPS2* is an ABA-inducible and dehydration-responsive gene which plays an important role in stress tolerance in chickpea (Kaur *et al.*, 2008, 2013). However, the role and regulation of IMP in chickpea is unknown. Chickpea is the second most important pulse crop in the world and is mainly grown in arid and semi-arid regions. Chickpea faces various environmental stresses during its life cycle and has been considered as a rich source of tolerant genes for a wide range of abiotic stresses such as drought, cold, and salinity. Chickpea is considered to be a highly important crop for developing countries like India since it provides cost-efficient protein-rich nutrition for the human diet (Singh *et al.*, 1998; Ahmad *et al.*, 2005). Despite being an important food crop and a rich source of stress-tolerant genes, research in chickpea has always been restricted, possibly due to the non-availability of mutant resources and a lack of efficient and dependable transformation protocols.

In this work, the IMP coding gene and the corresponding cDNA has been isolated and cloned from chickpea. Further, substrate specificity and detailed enzymatic properties have been studied using bacterially expressed and purified enzyme protein. Expression of the *IMP* gene together with *MIPS* genes has been investigated. For functional studies, complementation experiments have been carried out in *Arabidopsis* IMP (*vtc4*) mutants. Over-expression lines for *CaIMP* have also been generated in *Arabidopsis* to investigate its role in plant stress adaptation.

## Materials and methods

### Plant material, growth conditions and treatments

Chickpea (*Cicer arietinum*) cv. BGD72 was used in the present study and was obtained from the Indian Agricultural Research Institute (IARI), New Delhi, India. Seeds were germinated and grown according to the method described by Boominathan *et al.* (2004).

Six-day-old seedlings were exposed to various stresses for 5h as described previously by Boominathan *et al.* (2004). For hormone treatment, 6-d-old seedlings were treated with 100 μM ABA (abscisic acid), IAA (indole acetic acid), SA (salicylic acid), and GA (gibberellic acid) (Sigma) for 5h as described earlier (Kaur *et al.*, 2013).

*Arabidopsis thaliana* ecotype Columbia-0 and *vtc4* mutants (vtc4-3:SAIL_843_G10 and vtc4-4:SALK_077222) were used in this study. *Arabidopsis* plants were grown in a 3:1 mixture of agropeat and vermiculite in a growth room under controlled conditions (16/8h light/dark cycle at 22±2 ºC and light intensity of 200 μmol photons m^–2^ s^–1^) for general growth and seed harvesting unless otherwise mentioned.

### *Isolation and molecular cloning of the* IMP *gene and cDNA from chickpea*

Total RNA was isolated from chickpea seedlings using the Tri reagent (Sigma) according to the manufacturer’s protocol and cDNA was made using Superscript III reverse transcriptase (Invitrogen) and the oligo dT anchor primer. Degenerate primers were designed based on the most conserved domain of IMP proteins and were used for 3′ RACE. Subsequently, 5′ RACE was performed based on the partial cDNA sequences using a kit (Invitrogen). The full-length cDNA for *CaIMP* was amplified from total cDNA using gene-specific primers. The amplicon was cloned into a pJET vector (Fermentas) and subsequently sequenced.

To achieve genomic sequences of *CaIMP*, PCR was performed using total chickpea genomic DNA. The amplified PCR product was cloned into a pJET vector (Fermentas) and sequenced. Primers are listed in Supplementary Table S1 at *JXB* online.

### Molecular cloning of the promoter region of *CaIMP*

Genomic DNA containing the putative promoter region of *CaIMP* was amplified from chickpea genomic DNA through genome walking using a genome walker kit (Clonetech). Genome walking PCR was performed on the genomic DNA library using gene- specific primers and the adaptor primer (AP1) provided in the kit. The resulting PCR products were re-amplified with nested gene-specific primers and the adaptor primer (AP2) provided in the kit. Amplified fragments were then cloned into a pJET vector (Fermentas) and sequenced.

### Quantitative real-time-PCR (qRT-PCR)

Quantitative real-time PCR was performed according to the procedure described by Kaur *et al.* (2008). Total RNA from different organs and seedlings of chickpea or *Arabidopsis* was isolated using the TRI reagent (Sigma) following the manufacturer’s instructions. cDNA was synthesized from cDNA synthesized from DNaseI (Ambion) treated 2 μg RNA using random primers following the manufacturer’s instructions (Applied Biosystems). Real-time PCR reactions were carried out on an ABI Step One real time PCR using primer pairs for *CaIMP* and 18S. The expression of *CaIMP* mRNA was normalized to the expression of 18S mRNA. For each real-time PCR reaction, 20 μl of a mixture containing 2 μl of diluted RT product, 10 μl of power SYBR green PCR master mix (Applied Biosystems), and 20 pmol of each forward and reverse primer was used. A negative control lacking the RT enzyme was also included in each assay. All reactions were performed in three biological replicates.

### Bacterial over-expression and purification of *CaIMP*

The cDNA for *CaIMP* was subcloned into the *Nde*I/*Xho*I sites of the expression vector pET-23b (Novagen). The resulting plasmid was introduced into the host strain *E. coli* BL-21 (DE3) (Sambrook and Russell, 2001). Transformed *E. coli* cells were grown in LB (Luria-Bertani) medium at 37 °C up to *A*_600_ of 0.5 absorbance units and induced by 0.5mM IPTG for 6h. The bacterial cells were collected by centrifugation and lysed by sonication in a buffer containing 20mM TRIS-HCl, pH 7.5, 10mM β-mercaptoethanol (βME), with a bacterial protease inhibitor cocktail (Sigma). The extracts were analysed by 12% SDS-PAGE (Laemmli, 1970).

Solubilization and purification of the recombinant protein was performed according to the procedure described by Majee *et al.* (2004). The dialysed expressed protein sample was purified using a nickel charged affinity columns (GE Healthcare) following the manufacturer’s protocol and used for further analysis.

### Protein extraction, partial purification, and IMP assay

Seedlings of chickpea and *Arabidopsis* were ground to a fine powder with liquid nitrogen in a pre-chilled mortar and pestle. Two millilitres of chilled extraction buffer, containing 50mM TRIS-Cl (pH 8), 20mM KCl, 10% glycerol, 10mM βME, and 1% plant protease inhibitor cocktail (Sigma) was added to 0.5g of tissue powder and homogenized. The homogenate was then centrifuged for 15min at 12 000 *g* at 4 ºC.

For partial purification of the crude protein, the protein supernatant was centrifuged in a Millipore centrifugal filter device (Amicon Ultra) at 7500 *g* for 10min at 4 °C in order to remove low molecular weight compounds below 10kDa. The purified protein was used for determining IMP activity.

Protein concentration was determined according to Bradford (1976) using bovine serum albumin as standard.

IMP enzyme activity was assayed by the colorimetric estimation of released inorganic phosphate (Pi) as described by Baykov *et al.* (1988). A typical 100 μl reaction mixture contained 30mM substrate and 3mM MgCl_2_ in 50mM TRIS-Cl (pH 8) buffer. The reaction was carried out at 37 °C for 1h using ~10 μg of purified enzyme or 25 μg partially purified proteins. After incubation, 700 μl of deionized water and 200 μl of malachite green solution were added to develop the colour and the released inorganic phosphate was subsequently determined spectrophotometrically at *A*_630_.

### *Arabidopsis thaliana* transformation and transgenic analysis

To generate over-expression lines of *CaIMP* in *Arabidopsis thaliana* (Col-0), the full-length *CaIMP* cDNA was cloned into the plant expression vector pCAMBIA 2301 under the control of the CaMV 35S promoter for constitutive expression. Transgenic plants were obtained by *Agrobacterium*-mediated transformation using the floral dip method (Clough and Bent, 1998). For the complementation study, *CaIMP* was cloned into the plant expression vector pCAMBIA 1301 under the control of the 35S promoter and *vtc4* mutants were used for transformation. Transformed plants were selected by hygromycin resistance followed by PCR analysis.

### Gas liquid chromatography (GLC) analysis

Frozen seedlings and tissues were ground into powder and were homogenized with a mixture of methanol, water, chloroform, and trichloro acetic acid (12:5:2:1 by vol.) for the isolation of total water-soluble sugars and polyols. The aqueous phase containing the bulk of the water-soluble plant metabolites was lyophilized to complete dryness.

For GLC analysis, the lyophilized samples were transferred quantitatively to glass reaction vials in pyridine and evaporated under high vacuum. Samples were kept in a vacuum desiccator for 24h over P_2_O_5_ for the complete removal of water before being TMS-derivatized with Tri-Sil- Z (Pierce) and run through a Chemito 1000 GC equipped with a flame ionization detector for gas liquid chromatography. Quantitation was done using specific Clarity Lite software. The GLC column used was a BP-1 megabore capillary column obtained from SGE, Australia that was 30 m long and 0.53mm i.d. The oven temperature was programmed from 200 ºC to 280 ºC at 4 ºC min^–1^. The initial and final temperatures were kept isothermal for 2min and 10min, respectively. The injection port and detector temperatures were 300 ºC and 350 º, respectively. Nitrogen gas was used as the carrier with a flow rate of 10.06ml min^–1^. The quantification of *myo*-inositol was made against similar runs with an authentic standard.

### Total ascorbate content

Tissue (0.2g) was homogenized in 2 vols of chilled 5% meta-phosphoric acid, the homogenate centrifuged at 20 000 *g* for 15min at 4 ºC, and the supernatant was collected. Ascorbate was quantified by the reduction of Fe^3+^ to Fe^2+^ and the detection of a Fe^2+^ complex with 2,2′-dipyridyl as described by Kampfenkel *et al.* (1995).

### Germination assay and cold sensitivity

Wild-type, transgenic, and mutant lines were grown in identical controlled conditions as described earlier (see section on Plant material, growth conditions, and treatments). Seeds from mature brown siliques were harvested on the same day from all plants and then stored in the dark under dry conditions at room temperature (24±2 °C) for further use. The germination assay was carried out as described previously (Verma *et al.*, 2013). Triplicate samples of 100 age-matched seeds of *Arabidopsis* were used for the germination assay. Seeds were surface-sterilized, plated on to half-strength Murashige and Skoog (MS) agar media (Sigma), and kept in the dark at 4 °C for 3 d before being allowed to germinate in controlled culture room conditions (22±2 ºC with a 16/8h light/dark cycle). To evaluate germination under stress conditions, seeds were plated on half-strength MS agar media supplemented with NaCl (150mM), PEG (–0.5MPa), or paraquat (0.5 μM) and were incubated at 37 °C for 2 d for the heat treatment.

Seed germination was determined to be completed when the radicle protruded beyond the testa and was assessed for 7 d or as mentioned in the legend. In all experiments, at least three independent transformant lines were evaluated.

For the analysis of cold sensitivity of the *vtc4* mutants, seeds of the *vtc4* mutants, along with complemented lines, the wild type, and vector controls were surface-sterilized and then plated on half-strength MS agar media and germination assessed and root length measurements taken at 22 °C and 10 °C.

For root length measurements, plates were placed vertically and initially grown at 22 °C for 3 d in a growth room. The root length was marked and plates were divided into two sets; one set was continued at 22 °C and the other set was transferred to 10 °C. After 16 d, the root lengths were measured and compared.

### Stress treatments and seedling growth

Seeds from all genotypes were germinated on normal half-strength MS-agar plates and kept in a growth room under controlled conditions. Seven-day-old seedlings were transferred to half-strength MS-agar media supplemented with NaCl (150mM), PEG (–0.5MPa) or paraquat (1 μM) and kept in a growth room at 22 °C. For cold and heat treatment plates were kept at 4 °C and 37 °C, respectively. These plates were monitored daily for the observation of stress-induced changes. For analysing the antioxidant potential and quantitative stress profiling of different lines of *Arabidopsis*, seedlings were given different stress treatments. Aqueous solutions of 1 μM paraquat, 150mM NaCl or –0.5MPa PEG were applied to seedlings for 3 d at 22 °C. Cold and heat treatment was given by transferring seedlings to 4 °C and 37 °C, respectively. These stressed seedlings along with controls were used for estimating MDA content, H_2_O_2_ content, chlorophyll content, and biomass.

### Hydrogen peroxide content (H_2_O_2_)

Hydrogen peroxide content was measured according to the method of Alexieva *et al.* (2001). Seedlings (0.2g) were homogenized in 2ml of 0.1% (w/v) trichloroacetic acid (TCA) and were centrifuged at 13 000 *g* for 15min at 4 ºC. Supernatant (0.5ml) was used in the reaction mixture that consisted of 0.5ml of 10mM potassium phosphate buffer (pH 7.0) and 1ml of 1M potassium iodide (KI) reagent and was incubated for 1h in the dark to develop colour. Finally, the absorbance was measured at 390nm. The amount of H_2_O_2_ was calculated using a standard curve prepared from known concentrations of H_2_O_2_.

### Malondialdehyde (MDA) content

The procedure described by Heath and Packer (1968) was followed for measuring the MDA content. 0.2g of seedlings were homogenized in 2ml of 0.25% (w/v) 2-thiobarbituric acid prepared in 10% (w/v) trichloroacetic acid. The homogenate was incubated at 95 ºC for 30min and centrifuged at 13 000 *g* for 30min. Absorbance of the supernatant was measured at 532nm and at 600nm. The concentration of MDA was calculated by using an extinction coefficient of 155mM^–1^ cm^–1^.

### Chlorophyll and carotenoid content

Seedlings (0.2g) were ground in liquid nitrogen and pigments extracted using 2ml chilled 80% acetone. The homogenate was centrifuged at 13 000 *g* for 5min. Absorption of the supernatant was measured at 663, 645, and 470nm using a UV/VIS spectrophotometer. The concentrations of chlorophyll and carotenoids were determined according to the equations reported by Lichtenthaler and Wellburn (1983).

### Statistical analysis

Analysis of variance (ANOVA) followed by Duncan’s multiple comparison tests were used to determine statistically significant difference among the different genotypes and treatments. All the differences mentioned in the text were significant at *P* <0.05 or *P* <0.01.

## Results

### IMP activity is up-regulated under abiotic stress conditions

**Fig. 1.**
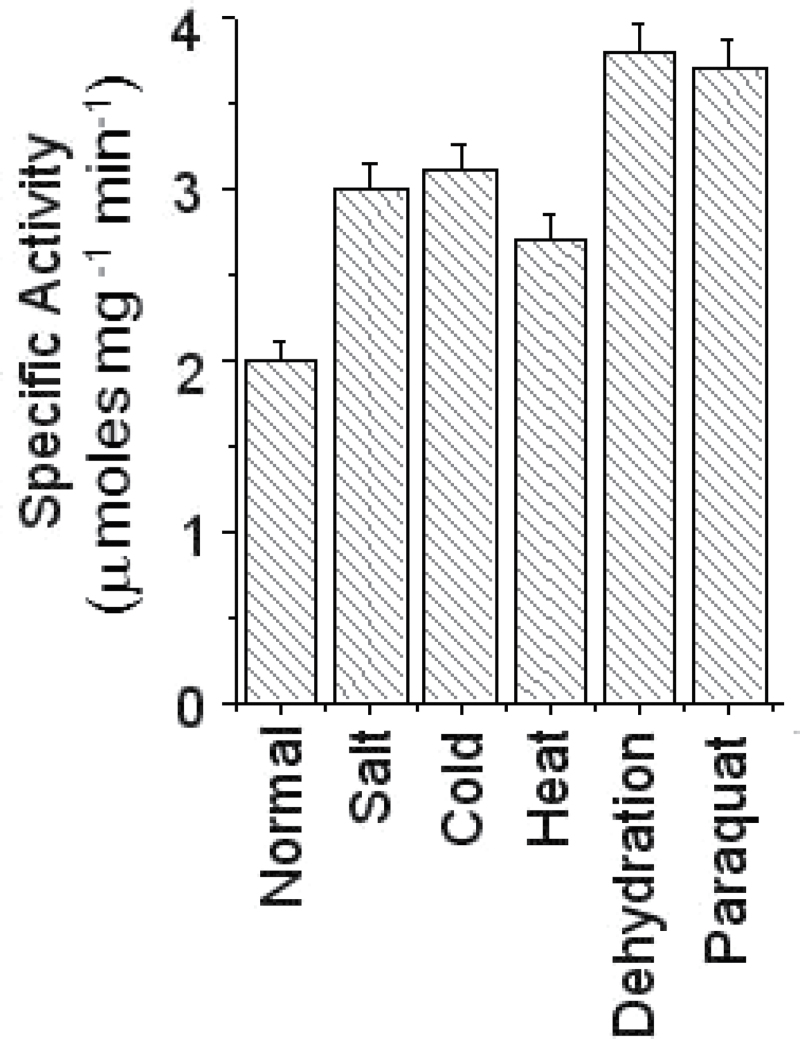
Enzymatic activity of inositol monophosphatase (CaIMP) in chickpea seedling under various abiotic stress conditions. IMP activity was determined in chickpea seedlings challenged with various stresses. ~25 μg of partially purified protein was used in each case. Specific activity was calculated as μmol Pi released mg^–1^ protein min^–1^. In each case values are mean ±SE of three replicates.

In response to adverse environmental factors, few stress-tolerant plants, including chickpea, have been reported to accumulate increased levels of *myo*-inositol for their adaptation to stress (Ishitani *et al.*, 1996; Boominathan *et al.*, 2004). Similarly, the activity of the first and rate-limiting enzyme MIPS has also been shown to be up-regulated in stress conditions (RayChaudhuri and Majumder, 1996; Majee *et al.*, 2004). However, it remains uncertain whether IMP activity is regulated by environmental cues or not. To explore this, IMP activity in chickpea seedlings challenged with various abiotic stresses has been examined here. Protein was extracted and partially purified from normal as well as various stress-treated seedlings and IMP activity was subsequently determined as described by previous studies (Baykov *et al.*, 1988; Patra *et al.*, 2007). The data revealed that seedlings subjected to abiotic stresses showed higher IMP activity compared with normally grown seedlings (Fig. 1), although the stress-induced up-regulation of IMP activity varied with different stresses. This result clearly indicates the possible involvement of IMP in inositol-mediated stress adaptation in chickpea.

### Molecular cloning and characterization of CaIMP

**Fig. 2.**
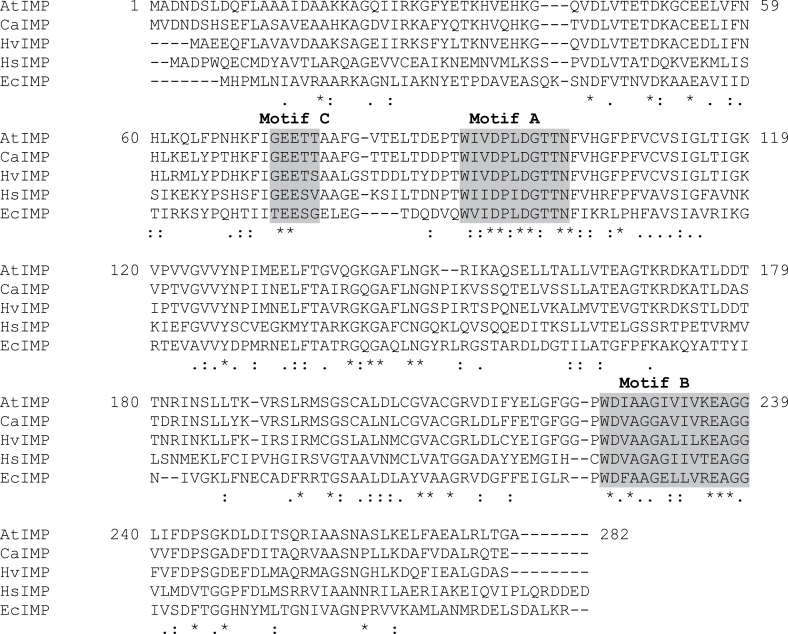
Multiple alignments of IMP protein sequences from eukaryotic and prokaryotic sources using the CLUSTAL-W multiple alignment program. Three characteristic signature motifs (Motif ‘A’ - DPIDGT, Motif ‘B’ - WDXAAG, and Motif ‘C’ - GEES) of the phosphatase super family is highlighted. The key amino acids in substrate and metal binding and nucleophile activation (i.e glutamate [E]^73^, aspartate [D]^94^, leucine/isoleucine [L/I]^96^, aspartate [D]^97^, glycine [G]^98^, threonine/serine [T/S]^99^, and aspartate [D]^225^) are underlined. [AtIMP, *Arabidopsis thaliana* IMP; CaIMP, *Cicer arietinum* IMP; HvIMP, *Hordeum vulgare* IMP; HsIMP, *Homo sapiens* IMP; EcIMP, *Escherichia coli* IMP.]

A pair of degenerate primers based on the conserved domain of IMP protein sequences was used for 3′ RACE. Based on this partial sequence, 5′ RACE was successfully performed and eventually a full-length cDNA (*CaIMP*) was amplified. The amplified product was cloned into a pJET vector and sequenced. The sequence was submitted to the NCBI GenBank database with the accession number JX069956. The full-length composite sequence including the 5′ and 3′ untranslated regions is 1087bp in length and contains an open reading frame of 813bp encoding a polypeptide of 270 amino acids. The polypeptide sequence possesses three characteristic signature motifs (Motif ‘A’ –DPIDGT, Motif ‘B’- WDXAAG, and Motif ‘C’-GEES) of the lithium-sensitive phosphatase super family enzymes including IMP, inositol polyphosphate 1-phosphatase, and fructose 1,6-*bis*phosphatase (Neuwald *et al.*, 1992; Atack *et al.*, 1995). The key active-site amino acids responsible for substrate binding, metal interaction, and nucleophilic activation interactions in IMP are also found to be conserved in the CaIMP protein sequence (Fig. 2). A comparison of the CaIMP protein sequence with other IMPs was analysed and CaIMP showed 75–89% identity with IMPs from other plant sources, 45% with human IMP, and 40% with *E. coli* IMP (Fig. 2).

To analyse the genomic structure of *CaIMP*, specific primers were designed based on the sequence of corresponding cDNA and PCR was subsequently performed with total genomic DNA. The organization of this gene is illustrated in Supplementary Fig. S1 at *JXB* online. The genomic structure revealed that the *CaIMP* gene spans about 4613bp in length and contains 12 exons and 11 introns (accession no. JX069957).The sequences at the exon–intron boundaries follow the GT–AG rule. Similar genomic structure, i.e. 12 exons and 11 introns were also reported in the case of *Arabidopsis* (*vtc4*) and tomato *IMP* (*LeIMP1*) while *IMP* from barley and rice were shown to have 10 exons and 9 introns (Fu *et al.*, 2008).

### CaIMP exhibits broad substrate specificity

**Fig. 3.**
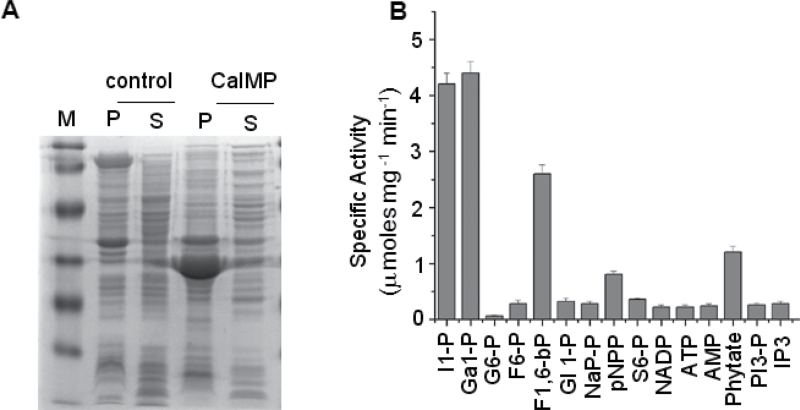
Biochemical characterization of CaIMP. (A) 12% SDS PAGE analysis of CaIMP over-expressed protein in *E. coli* BL21. [Control, pET23d empty vector transformed induced cells; CaIMP, *CaIMP* transformed induced cells; M, molecular weight marker; P, pellet fractions; S, soluble fractions.] (B) Substrate specificity of purified recombinant CaIMP protein. Substrate specificity was determined using all the substrates at a final concentration of 30 μM and at 37 °C. [I1-P, d-*myo*-inositol 1-phosphate; Ga1-P, l-galactose 1-phosphate; G6-P, α-d-glucose 6-phosphate; F6-P, d-fructose 6-phosphate; F1,6-bP, d-fructose 1,6-*bis*phosphate; Gl1-P, β-glycerol 1-phosphate; NaP-P, sodium pyrophosphate; pNPP, *p*-nitrophenyl phosphate; S6-P, d-sorbitol 6-phosphate; NADP, β-nicotinamide adenine dinucleotide phosphate; ATP, adenosine triphosphate; AMP, adenosine monophosphate; PI3-P, phosphotidyl inositol triphosphate; IP3, inositol triphosphate.] In each case values are mean ±SE of three replicates. In each experiment, 10 μg purified protein was used. Specific activity was calculated as μmol Pi released mg^–1^ protein min^–1^.

Apart from *myo*-inositol 1-phosphate as substrate, IMP was shown to have varied substrate specificities in different organisms (Parthasarathy *et al.*, 1997; Stec *et al.*, 2000; Torabinejad *et al.*, 2009). Hence, there was a need to examine the substrate specificity and other enzymatic properties of the CaIMP protein. To determine this, *CaIMP* cDNA was cloned into a pET-23b bacterial expression vector and induced by IPTG as described in the Materials and methods section. CaIMP was expressed as a ~30kDa protein predominantly in the particulate fraction as determined by SDS-PAGE analysis (Fig. 3A). Subsequently, the particulate fraction was solubilized in 8M urea buffer as described by Majee *et al.* (2004). The expressed and solubilized CaIMP protein was purified to near homogeneity through nickel charged affinity chromatography and then used to determine the enzyme’s characteristics. Purified active fractions were initially used to test the substrate specificity with various sugar phosphates including the general substrate *myo*-inositol 1-phosphate and a few other phosphorylated organic compounds. Results showed that the enzyme could primarily hydrolyse *myo*-inositol 1-phosphate and l-galactose 1-phosphate and thus indicated its possible participation in *myo*-inositol and ascorbate biosynthesis. Interestingly, the enzyme also showed substantial activity on fructose 1,6-*bis*phosphate and considerable activity on phytate. However, the enzyme failed to hydrolyse IP3, PIP3, NADP, ATP, AMP, glucose 6-phosphate, and glycerol 6-phosphate (Fig. 3B). To examine the requirement of the divalent cation for the activity of CaIMP, IMP activity was checked in the presence of several divalent cations (5mM each) and the maximum activity was observed in the presence of Mg^2+^ (see Supplementary Fig. S2 at *JXB* online).

Since the predominant activity was observed on *myo*-inositol 1-phosphate and l-galactose 1-phosphate, there was a need to compare the *K*_m_ and *V*_max_ values for both the substrates. The enzyme showed nearly same *K*_m_ values for *myo*-inositol 1-phosphate and l-galactose 1-phosphate suggesting fairly similar affinity towards both the substrates. For both substrates, the optimum temperature for enzyme activity was 40 °C and the optimum pH was 8.0 (see Supplementary Fig. S3A, B at *JXB* online). It was also observed that the activity for both substrates increased in the presence of Mg^2+^ and was found to be maximum at 5mM Mg^2+^. These data are summarized in Table 1.

**Table 1.** Biochemical characterization of recombinant CaIMP

Characters	CaIMP	
Substrates	*myo*-inositol 1-phosphate	l-galactose 1-phosphate
*K*_m_ (mM)	0.0259	0.0167
*V*_max_ (μmol min^–1^ mg^–1^)	4.86	5.61
Temp optimum (°C)	40 °C	40 °C
pH optimum	8	8
MgCl_2_ (mM)	5	5

The IMP protein belongs to the lithium-sensitive phosphatase superfamily of enzymes (Neuwald *et al.*, 1992) and, therefore, it was necessary to check the inhibition of the catalytic activity of IMP by the monovalent cation Li^+^ along with other monovalent cations. As shown in Supplementary Fig. S4 at *JXB* online, both *myo*-inositol monophosphatase and l-galactose 1-phosphatase activities are indeed inhibited by Li^+^, while other monovalent cations like Na^+^, K^+^, and 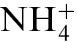 had no significant effect on CaIMP. Our biochemical study clearly demonstrated that CaIMP is a lithium-sensitive phosphatase enzyme with broad substrate specificity.

### CaIMP is differentially expressed in chickpea

In response to dehydration stress, the increased production of *myo*-inositol as a consequence of co-ordinate transcriptional induction of the MIPS gene has been reported in chickpea (Boominathan *et al.*, 2004; Kaur *et al.*, 2008). In addition, the *CaMIPS2* gene was found to be ABA-inducible and dehydration-responsive unlike *CaMIPS1* (Kaur *et al.*, 2013). However, under such a situation, the expression pattern of the gene encoding IMP which essentially produces free inositol from *myo*-inositol monophosphate has not been investigated. Thus, there was also a need to examine whether or not *CaIMP* is co-ordinately induced under such conditions. To investigate this, chickpea seedlings were subjected to various abiotic stress treatments and RNA was extracted. The pattern of mRNA accumulation of the IMP gene along with MIPS genes *CaMIPS1* and *CaMIPS2* was subsequently analysed using qRT-PCR as described in the Materials and methods.

**Fig. 4.**
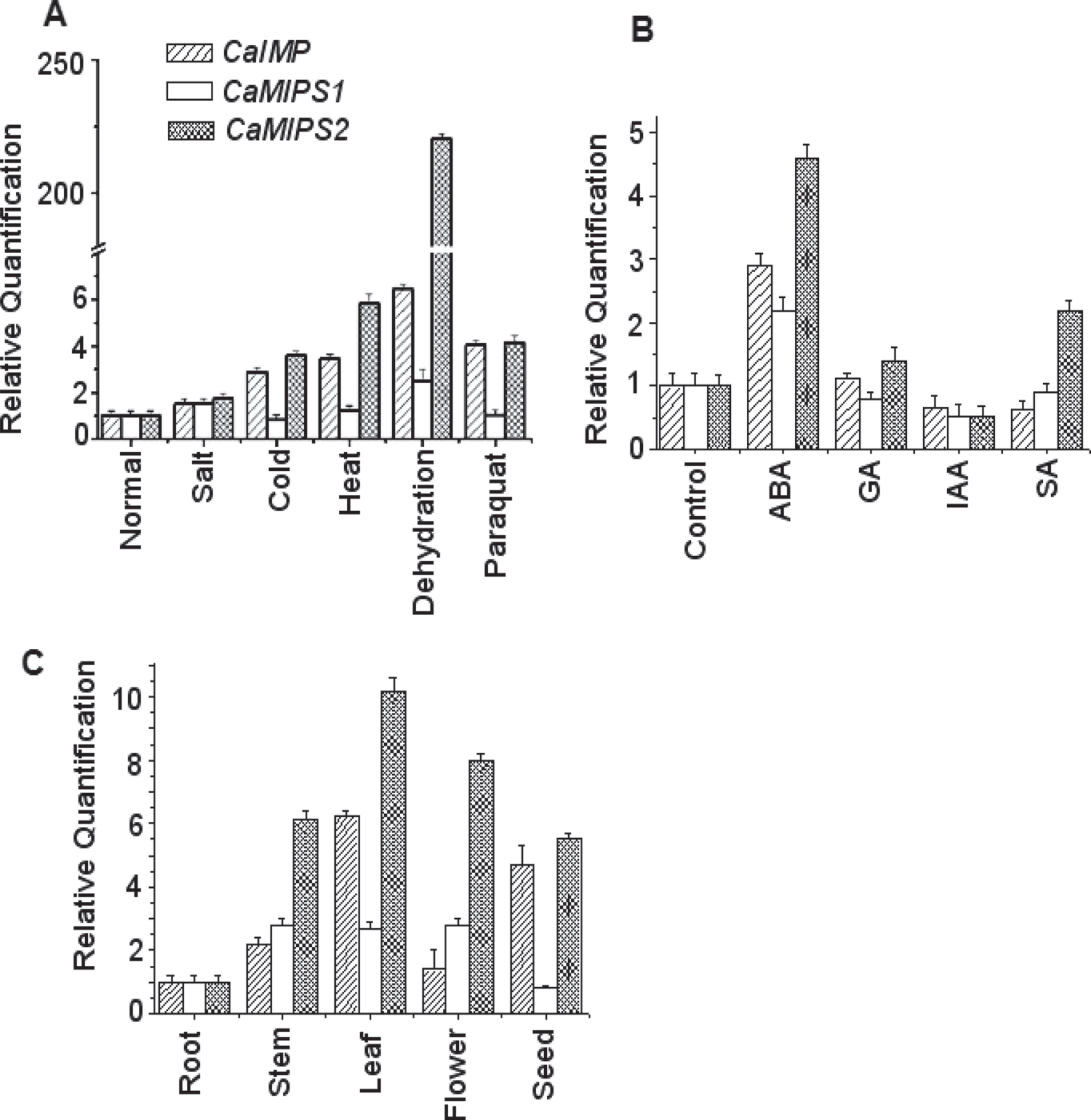
Quantitative RT PCR analysis of *CaIMP*, *CaMIPS1*, and *CaMIPS2* (A) under different abiotic stresses, (B) in the presence of exogenous hormones, and (C) in different organs. Total RNA from each sample was reverse transcribed and subjected to real-time PCR analysis. The relative expression value of each gene was normalized to an endogenous control 18S and calculated using the ΔΔCT method (Applied Biosystems). Values are the result of triplicate analysis of three biological replicates. Error bars indicate the standard deviation.

As reported previously, the *CaMIPS2* transcript was definitely increased under most of the stress conditions although, with the dehydration stress, transcript accumulation was enhanced drastically whereas *CaMIPS1* expression was not greatly altered under similar conditions. Similar to *CaMIPS2*, the *CaIMP* transcript was also found to be increased in all stresses with maximum expression with dehydration stress, although the level of induction was comparatively lower than *CaMIPS2* (Fig. 4A). To investigate further, the expression pattern of these *myo*-inositol biosynthetic genes in the presence of exogenous hormones was analysed. The result showed that, like *CaMIPS2,* the *CaIMP* transcript was also increased following ABA treatment, whereas the *CaMIPS1* transcript was more or less unaltered. In SA-treated seedlings, only the *CaMIPS2* transcript level was found to be increased compared with water-treated control seedlings (Fig. 4B).

Next, in order to investigate the spatial expression patterns of *CaIMP* and the *CaMIPS* genes *(CaMIPS1* and *CaMIPS2*), in different organs in chickpea, RNA was extracted from the roots, stems, leaves, flowers, and seeds and qRT-PCR was conducted. Data revealed that *CaMIPS1,CaMIPS2*, and *CaIMP* transcripts were observed at different levels in different organs although the maximum *CaIMP* transcript was observed in leaves (Fig. 4C).

In conclusion, *CaIMP* exhibited differential transcript accumulation in different organs, with different stresses and hormones and followed a fairly similar pattern of expression as *CaMIPS2*, particularly under stress conditions.

In addition, in order to understand the molecular basis of the stress-induced expression of *CaIMP*, 5′ upstream regulatory sequences were amplified through genome walking as described in the Materials and methods. Subsequently, a putative promoter sequence was screened for *cis*-acting regulatory elements using various programs like PLACE (Higo *et al.*, 1999), CISTER or PlantCARE database (Lescot *et al.*, 2002) and various *cis*-regulatory sequences like phosphate starvation, light, sugar, and various hormone- responsive elements were observed. In addition, a range of *cis*-regulatory elements for drought, heat, and salt-stress-induced expression were identified.

Nucleotide sequences with *cis*-regulatory element are shown in Supplementary Fig. S5 at *JXB* online. Interestingly, the CRT/DRE element is present in both the *CaIMP* and *CaMIPS2* promoters but is absent in the *CaMIPS1* promoter (Kaur et al., 2008, 2013).

### CaIMP functionally complements *vtc4* in *Arabidopsis*

**Fig. 5.**
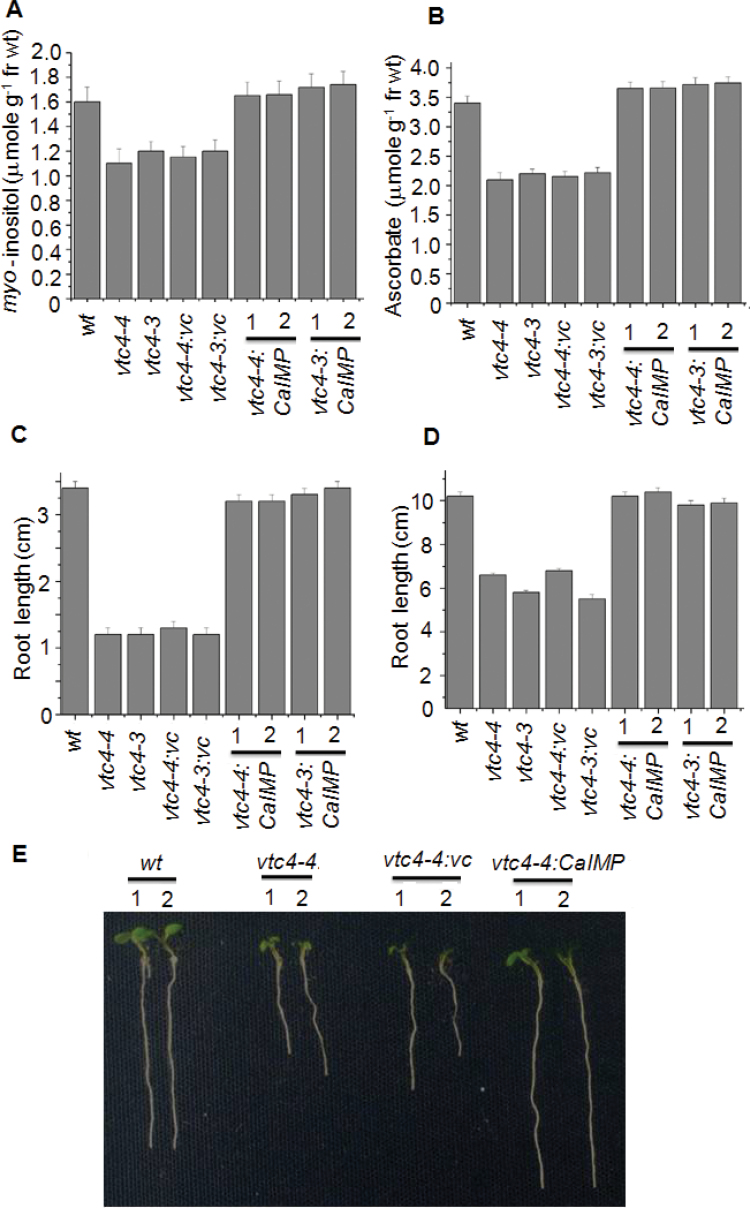

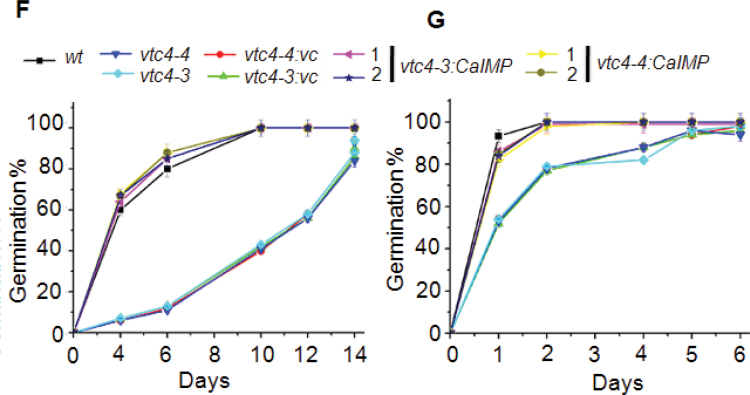
Characterization of *vtc4* mutants (*vtc4-4*:SALK_077222 and *vtc4-3*: SAIL_843_G10) complemented by *CaIMP*. (A) *myo*-Inositol content and (B) ascorbate content of wild type (*wt*), *vtc4* mutants (*vtc4-4* and *vtc4-3*), *vtc4* mutants harbouring empty vector (*vtc4-4:vc* and *vtc4-3:vc*) and complemented lines (*vtc4-4:CaIMP* lines 1 and 2 and *vtc4-3:CaIMP* lines 1 and 2) of *Arabidopsis thaliana*. Inositol and ascorbate content were measured in seedlings. (C, D) Root growth assay at (C) 10 °C and (D) 22 °C of wild type (*wt*), *vtc4* mutants (*vtc4-4* and *vtc4-3*), *vtc4* mutants harbouring empty vector (*vtc4-4:vc* and *vtc4-3:vc*) and complemented lines (*vtc4-4:CaIMP* lines 1 and 2 and *vtc4-3:CaIMP* lines 1 and 2) of *Arabidopsis thaliana*. (E) Photograph showing the representative phenotype comparison of 10-d-old wild type (*wt*), *vtc4-4*, *vtc4-4:vc,* and *vtc4-4:CaIMP* lines 1 and 2 of *Arabidopsis thaliana* grown at 10 °C. (F, G) Germination assay at (F) 10 °C and (G) 22 °C of wild type (*wt*), *vtc4* mutants (*vtc4-4* and *vtc4-3*), *vtc4* mutants harbouring empty vector (*vtc4-4:vc* and *vtc4-3:vc*) and complemented lines (*vtc4-4:CaIMP* lines 1 and 2 and *vtc4-3:CaIMP* lines 1 and 2) of *Arabidopsis thaliana.* Surface-sterilized seeds of all genotypes were allowed to germinate on half-strength MS agar plates and grown at 10 °C (cold) and 22 °C (RT) (as mentioned in the Materials and methods section). Germination was also scored until 7 and 14 d after transferring to 22 °C and 10 °C, respectively. In each case values are mean ±SE of three replicates.

Our biochemical study demonstrated that CaIMP predominantly hydrolyses *myo*-inositol 1-phosphate as well as l-galactose 1-phosphate and further suggests its possible participation in *myo*-inositol and ascorbate biosynthesis. To confirm this, a genetic complementation experiment was carried out where the *vtc4* knock-out mutants (*vtc4-3* and *vtc4-4*) were used. Earlier VTC4 (IMP) protein was shown to have both *myo*-inositol 1-phosphatase and l-galactose 1-phosphatase activity. Loss-of-function of the *vtc4* gene leads to a decrease in both the *myo-*inositol and ascorbate content in *Arabidopsis* plants with reduced root growth and germination rate (Torabinejad *et al.*, 2009). To complement this, CaIMP was ectopically expressed in the *vtc4-3* and *vtc4-4* mutants under the control of the 35S CaMV promoter. Transformed plants were selected on both kanamycin and hygromycin double selections plates. Subsequently, *myo*-inositol and ascorbate content were estimated as described in the Materials and methods. *myo*-Inositol and ascorbate contents in *vtc4* mutants transformed with *CaIMP* were found to be comparable with that of wild-type plants (Fig. 5A, B). Germination percentage as well as root growth responses were also measured at low (10 °C) and normal temperature (22 °C). As reported earlier by Torabinejad *et al.* (2009), under low temperature, both root growth and germination rate of the *vtc4* mutants (*vtc4-3* and *vtc4-4*) were found to be drastically reduced compared with the wild type (Fig. 5C, E, F;, see Supplementary Fig. S6 at *JXB* online). Similarly, under normal temperature, the difference in root length and germination rate was also observed between the *vtc4* mutants and the wild type (Fig. 5D, G). However, mutants transformed with *CaIMP* exhibited normal root growth and germination and replenished the deficiency of *myo*-inositol and ascorbate. By contrast, vectors without IMP could not repair these defects in the mutant plants and thus clearly established the involvement of CaIMP in *myo*-inositol and ascorbate biosynthesis.

### Over-expression of *CaIMP* in *Arabidopsis* results in enhanced myo-inositol and ascorbate content and improves seed germination and seedling growth under abiotic stress conditions

**Fig. 6.**
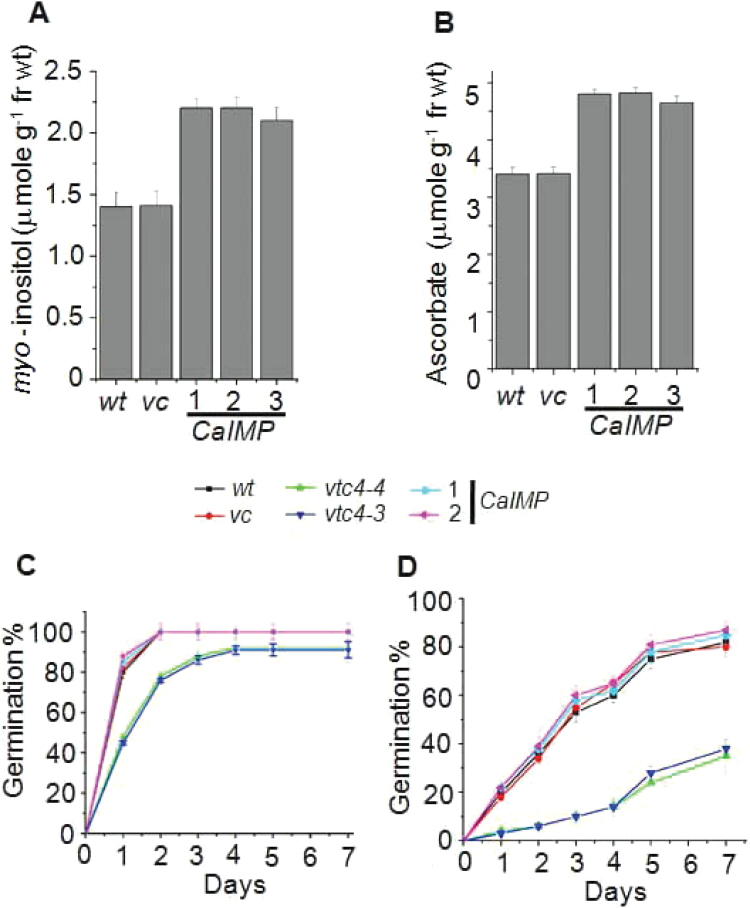

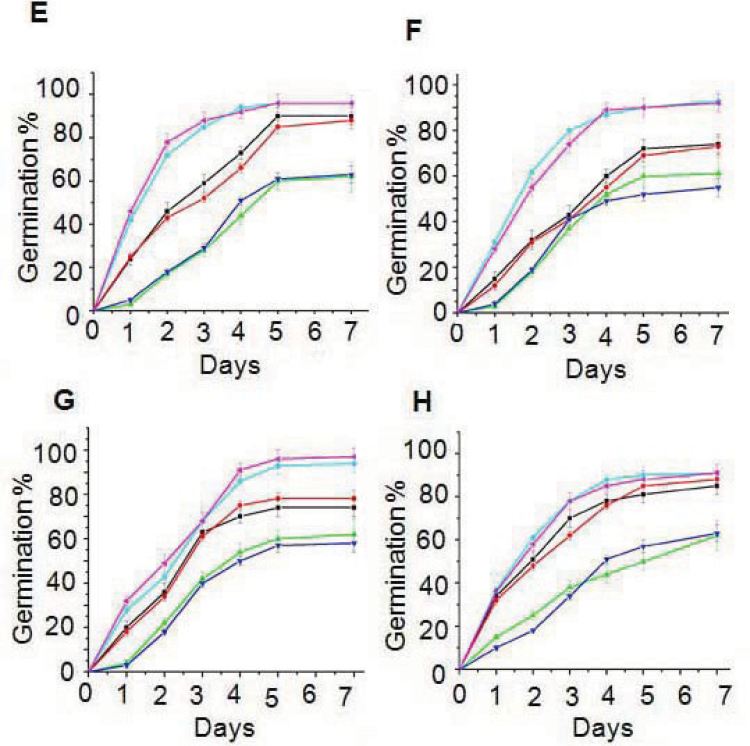
*CaIMP* transgenic lines exhibit improved seed germination. (A) *myo*-Inositol content, and (B) ascorbate content of wild type (*wt*), vector control (*vc*), and *CaIMP* over-expression lines (35S:*CaIMP* lines 1, 2, and 3). Inositol and ascorbate contents were measured in leaves as described in the Materials and methods section. In each case values are mean ±SE of three replicates. (C–H) Germination percentage of wild type (*wt*), vector control (*vc*)*, vtc4* mutants (*vtc4-4* and *vtc4-3*) and *CaIMP* over-expression lines under various stress treatments. Germination percentage at (C) normal, (D) cold (10 °C), (E) salinity (NaCl, 150mM), (F) dehydration (PEG, –0.5MPa), (G) oxidative (paraquat, 0.5 μM), and (H) heat stress (37 °C). Surface-sterilized age-matched seeds of all genotypes were plated on half-strength MS agar with or without supplementation for the required stress. Plates were kept at 4 °C for 3 d for stratification and then transferred to a growth room at 22±2 °C.Values are mean ±SE of three replicates.

Inositol and ascorbic acid were previously shown to play key roles in plant responses to abiotic stresses. In our earlier experiments, it was observed that *CaIMP* is implicated in both *myo*-inositol and ascorbic acid biosynthesis in plants. Hence, there was a need to evaluate the potential role of IMP in mitigating the adverse effect of abiotic stresses in plants. Furthermore, to check whether over-expression of *CaIMP* in *Arabidopsis* can enhance both *myo*-inositol and ascorbate accumulation, *CaIMP* was constitutively expressed in *Arabidopsis* under the control of the 35S CaMV promoter. *CaIMP*-transformed plants were initially screened for kanamycin resistance and GUS reporter gene expression. Finally, the presence of *CaIMP* in kanamycin resistant with GUS-expressing transgenic lines was confirmed by PCR analysis using *CaIMP* specific primers. The transgenic lines exhibiting a 3 kan^R^:1 kan^S^ segregation pattern in its progeny were selected and homozygous lines for the *CaIMP* gene were used for further analysis. Transcript expression of the transgene was analysed in several independent lines in the T_3_ generation and, compared with vector controls, a 10–20-fold higher transcript accumulation of CaIMP was observed. *myo*-Inositol and ascorbic acid content were then estimated in these selected transgenic lines and the enhanced accumulation of *myo*-inositol and ascorbate was observed in *CaIMP* transformed lines compared with the wild type or vector transformed lines (Fig. 6A, B). These observations were verified in multiple independent experiments and the responses to various abiotic stresses of the transgenic lines and control plants were compared. Initially, the effect of various stresses on seed germination was tested. The germination rate of the wild type, vector controls, and *CaIMP* transformed lines were similar when tested on normal MS media. However, the germination of wild-type seeds was found to be significantly inhibited on MS media supplemented with NaCl, paraquat, PEG, and at high temperature, whereas germination of *CaIMP* transformed lines was less affected in similar conditions. Under these adverse conditions, over-expression lines completed germination significantly earlier than the vector controls or wild type seeds (Fig. 6C–H).

In an earlier study, *vtc4* mutants were shown to have delayed seed germination and elevated sensitivity to NaCl and ABA (Torabinejad *et al.*, 2009). To confirm this role of IMP and to assess the germination rate in *vtc4* mutants under stress conditions, similar experiments were carried out on *vtc4* mutant lines. Even in normal MS media, mutant seeds took a longer time to complete germination compared with the wild type. In addition, in the presence of NaCl, PEG, paraquat, and high temperature, the inhibition of germination of *vtc4* mutants was more intense than the wild type (Fig. 6C–H). CaIMP could compensate for this reduced seed germination phenotype in complemented lines and further confirms this role of CaIMP in seed germination. These results clearly demonstrate that IMP plays an essential role in seed germination particularly under environmental stress conditions.

**Fig. 7.**
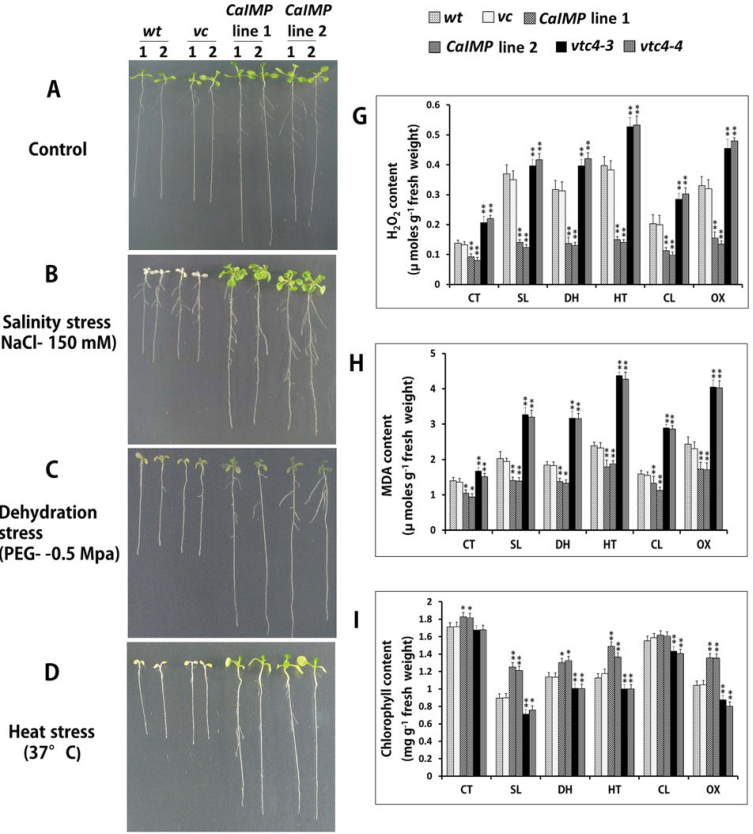

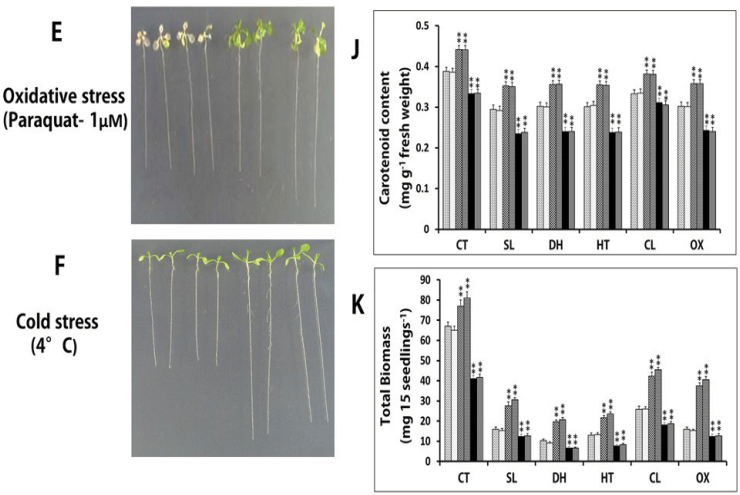
*CaIMP* transgenic lines exhibit improved seedling growth under abiotic stresses. (A–F) Comparative phenotype analysis of seedlings of wild type (*wt*), vector control (*vc*), and *CaIMP* over-expression lines (lines 1 and 2) under various abiotic stresses. Seven-day-old seedlings were subjected to various stress treatments (as described in the materials and methods section) and the growth pattern was compared. (A) Control conditions, (B) salinity stress (NaCl, 150mM), (C) dehydration stress (PEG, –0.5MPa), (D) heat stress (37 °C), (E) oxidative stress (paraquat, 1 μM), and (F) cold stress (4 °C). (G–K) Quantitative analysis of (G) H_2_O_2_ content, (H) MDA content, (I) chlorophyll content, (J) carotenoid content, and (K) biomass of wild type (*wt*), vector control (*vc*)*,CaIMP* over-expression lines (lines 1 and 2) and *vtc4* mutants (*vtc4-4 and vtc4-3*). Quantifications were done after various stress treatments in 14-d-old seedlings as described in the Materials and methods section. Values are mean ±SE of three replicates. [CT, control condition; SL, salinity; DH, dehydration; HT, heat; CL, cold; OX, oxidative stress.] Asterisks indicate significant difference between wild type and other genotypes: *^,^** *P*<0.05 and *P* <0.001, respectively.

To examine the role of CaIMP in seedling growth, particularly under environmental stress conditions, the growth pattern among wild type, vector controls, and over-expression lines were compared under various abiotic stresses. Seven-day-old seedlings were subjected to various abiotic stresses and the growth responses were evaluated. Under normal conditions, *CaIMP* over-expression lines, vector controls, and the wild type grew normally, although the root length of the *CaIMP* transformed lines was comparatively larger. However, under all stress conditions, the growth of the wild type and vector control lines was severely affected; root growth was inhibited and leaves turned yellow although the extent of the growth inhibition differed in different stress conditions. By contrast, *CaIMP* transformed seedlings were comparatively less affected and the growth performance was superior to that of the wild type and these transgenic seedlings remained green and healthy and root growth was comparatively less inhibited (Fig. 7A–F).

To assess this improved tolerance to stresses and to analyse antioxidant capacity, various parameters such as H_2_O_2_, MDA (as an index of lipid peroxidation), chlorophyll content, carotenoid content, and biomass were also measured in the wild type, vector controls, *CaIMP* over-expression lines, and *vtc4* mutants under normal and stress conditions (Fig. 7G–K), as these parameters have been successfully used to evaluate the physiological potential of stress tolerance in plants (Smirnoff,1995; Jiang and Zhang, 2002; Chagas *et al.*, 2008; Harb *et al.*, 2010; Saxena *et al.*, 2011).

Under all the stress treatments, the H_2_O_2_ and MDA contents were increased irrespective of genotypes, however, this increase was significantly higher (*P* < 0.05) in *vtc4* mutants followed by the wild type. The increment of H_2_O_2_ and MDA due to stress conditions was less pronounced in *CaIMP* over-expression lines than in the rest of the genotypes. Under stress situations, *vtc4* mutants accumulated H_2_O_2_ in the range of 0.3–0.6 μmol g^–1^ fresh weight, whereas the wild type or vector controls accumulated in the range of 0.2–0.4 μmol g^–1^ fresh weight (Fig. 7G). Noticeably, *CaIMP* over-expression lines limited the H_2_O_2_ content to a maximum of 0.2 μmol g^–1^ fresh weight. Similarly, under stress condition, *vtc4* mutants accumulated significantly higher MDA contents (3–4.5 μmol g^–1^ fresh weight) than wild type seedlings (1.5–2.5 μmol g^–1^ fresh weight). In the case of *CaIMP* over-expression lines, MDA accumulation was found to be limited to 1–1.5 μmol g^–1^ fresh weight (Fig. 7H). Chlorophyll and carotenoid contents were also compared among the genotypes (Fig. 7I, J). As expected, all the stress treatments led to a reduction in the chlorophyll and carotenoid contents in all the genotypes although the maximum decrease was recorded in NaCl-treated seedlings. However, the decrease was more profound in *vtc4* mutants while less so in over-expression lines. Similarly, compared with vector controls, *CaIMP* over-expression lines and *vtc4* mutants contained significantly higher and lower biomass, respectively under both normal and stress conditions (Fig. 7K). These biochemical analyses suggest that *CaIMP* over-expression lines were experiencing less damage under all stress treatments while, *vtc4* mutants were severely affected. Together these analyses clearly demonstrate that the *IMP* gene is implicated in both *myo*-inositol and ascorbic acid metabolism and plays an important role in seed germination and seedling growth, particularly under environmental stress conditions.

## Discussion

Besides an essential role in the *myo*-inositol metabolic pathway, IMP is reported to participate in various metabolic pathways like galactose metabolism in mammals, gluconeogenesis in methanogens, and ascorbate biosynthesis in *Arabidopsis* (Parthasarathy *et al.*, 1997; Stec *et al.*, 2000; Torabinejad *et al.*, 2009). The present study demonstrates that *CaIMP* is differentially expressed in chickpea and encodes a lithium-sensitive phosphatase enzyme with wide phosphate substrate specificity. Our data also indicate its participation in diverse multiple metabolic pathways. Evidence has been presented for the role of *CaIMP* in seed germination and seedling growth, particularly under adverse environmental conditions.

Chickpea IMP (CaIMP) amino acid sequences showed sequence homology with both prokaryotic and eukaryotic IMPs with characteristic IMP motifs (Fig. 2). More specifically, CaIMP possess motif A (amino acids region 87–100), B (amino acids region 219–233), and motif C (69–72) which contain all key amino acids involved in substrate, metal binding, and nucleophile activation. Importantly, these regions also occur in inositol polyphosphate 1-phosphatase and fructose 1,6-*bis*phosphatase enzymes (Atack *et al.*, 1995). Our biochemical study demonstrated that the enzyme has wide phosphate substrate specificity, but is clearly not a non-specific phosphatase, since the enzyme could not hydrolyse inositol triphosphate, NADP, ATP, AMP or glucose 6-phosphate (Fig. 3B). CaIMP exhibited fairly similar enzymatic properties with *myo*-inositol 1-phosphate and l-galactose 1-phosphate as the enzyme exhibited somewhat similar *K*_m_ and *V*_max_ values, pH optima, and temperature optima for both the substrates. Activities with both the substrates were found to be inhibited by lithium. A similar phenomenon has been described in *Arabidopsis* where VTC4 was shown to hydrolyse both *myo*-inositol 1-phosphate and l-galactose 1-phosphate (Torabinejad *et al.*, 2009). The enzyme activity of CaIMP on these two different substrates was further confirmed by genetic complementation study using *vtc4* mutants (Fig. 5). These combined biochemical and genetic data provided evidence for the role of CaIMP in l-galactose 1-phosphate and *myo*-inositol 1-phosphate hydrolysis and its involvement in *myo*-inositol and ascorbate biosynthesis in chickpea. However, unlike *Arabidopsis*, but similar to *Methanococcus jannaschii*, CaIMP was also found to hydrolyse fructose 1,6-*bis*phosphate which indicates its possible involvement in the gluconeogenesis pathway (Stec *et al.*, 2000). This fructose 1,6-*bis*phosphatase activity of the CaIMP enzyme has also been supported by our sequence analysis data. In addition, CaIMP was able to hydrolyse phytate which serves as a major phosphate store in seeds. Phytate was also shown previously to be hydrolysed by IMP in another legume plant *Glycine max* (Islas-Flores and Villanueva, 2007). However, interestingly, CaIMP was not able to hydrolyse sodium pyrophosphate and ATP, while soybean IMP could hydrolyse such substrates. These studies suggest that the substrate specificity of IMP proteins varies from species to species. Overall, our biochemical data clearly indicate that CaIMP is a multifunctional phosphatase enzyme and raise an intriguing possibility that this enzyme participates in diverse multiple metabolic pathways like *myo*-inositol, ascorbic acid, and gluconeogenesis. Our expression analyses indicate its possible role in stress adaptation, as *CaIMP* was up-regulated by various abiotic stresses. In addition, our qRT-PCR analyses also indicate the co-ordinate transcriptional activation of the *myo*-inositol biosynthetic genes *CaMIPS2* and *CaIMP* in response to abiotic stresses, particularly under dehydration stress (Fig. 4). The mRNA levels of the *myo-*inositol biosynthetic genes were found to be higher in leaves, seeds, and flowers than in the roots. These analyses indicate that *de novo* biosynthesis of *myo*-inositol is more active in photosynthetic and reproductive organs. Similarly, ascorbic acid biosynthesis is known to be more active in leaves and flowers. Subsequently, functional study clearly demonstrated that CaIMP participates at least in *myo-*inositol and ascorbate biosynthesis and plays an important role not only at the germination stage but also during subsequent seedling growth and development particularly under adverse environmental conditions (Figs 6, 7). Our biochemical data suggest the role of CaIMP in gluconeogenesis, however, further study is required to establish this finding. Earlier reports also indicate that both inositol and ascorbate play significant roles in seed germination and early seedling growth. A large amount of ascorbic acid is reported to utilize during the initial stage of seed germination and early seedling growth (Arrigoni *et al.*, 1997; Stasolla and Yeung, 2001). Various abiotic stresses like cold, dehydration, salt, and heat leads to the generation of reactive oxygen species (ROS) and causes lipid peroxidation and protein denaturation during germination thereby negatively affecting seed germination. Therefore, the ability of seeds to withstand different stresses is directly related to its ability to detoxify ROS (Vertucci and Farrant, 1995; Holmburg and Bulow, 1998; Dat *et al.*, 2000). There have been several reports of improving seed germination through enhancing antioxidant potential (Bailly, 2004) and ascorbate is one of the important antioxidants which eliminates ROS in cells. Similarly, *myo*-inositol is essential for the synthesis of galactinol, raffinose, and stachyose etc which play essential roles in seed biology and stress tolerance (Loewus and Murthy, 2000; Raboy, 2001). Apart from this, phytate, another substrate of this enzyme, is used during germination for the nourishment of the embryo as a source of mineral phosphorus, free inositol, and inositol phosphate as a signalling molecule (Raboy, 2003). The germination process has also been shown to be affected in the biosynthetic pathway mutants of *myo*-inositol and ascorbate, respectively (Clerkx *et al.*, 2004; Torabinejad *et al.*, 2009; Donahue *et al.*, 2010). Furthermore, gluconeogenesis also plays an important role during seed germination and post-germinative seedling growth. Active gluconeogenesis occurs in germinating seeds where this particular pathway converts fats and proteins to glucose for the synthesis of the polysaccharides, fructose and sucrose required for seedling growth and development (Graham, 2008). Our results have shown that CaIMP is implicated in these metabolic pathways which are likely to be necessary for seed germination. Apart from seed germination, the role of inositol and ascorbic acid in the stress tolerance of plants has long been recognized and well established. Inositol and its derivatives are implicated in stress tolerance through various ways such as protecting cellular structures from reactive oxygen species, by controlling turgor pressure or by acting as stress signalling molecules (Loewus and Murthy, 2000). Similarly, ascorbic acid is known to play a significant role in plant survival under stress conditions as the molecule has the ability to restrict the level of ROS and lipid peroxidation (Padh, 1990; Conklin, 2001; Schonhaf *et al.*, 2007) which is also evident in our study. In our study, it was also observed that the levels of H_2_O_2_ and MDA under stress conditions were significantly lower in *CaIMP* over-expression lines which had higher inositol and ascorbate contents than the wild type. By contrast, H_2_O_2_ and MDA were significantly higher in *vtc4* mutants which are more deficient in inositol and ascorbate than wild-type plants. Loss of chlorophyll and carotenoids due to stress were also less pronounced in over-expression lines while being more evident in knock-out mutants. The decrease in chlorophyll and carotenoid content was negatively associated with H_2_O_2_ and MDA content with increasing oxidative stress (Prochazkova *et al.*, 2001) and our results are also in agreement with previous studies. Together, it is clearly evident that at least *CaIMP* expression enhances the inositol and ascorbate contents of the plant and also indicates that these metabolic pathways are likely to be necessary for vigorous seed germination and early seedling growth, particularly under adverse environmental conditions. Our results also suggest that IMP plays an important role in root growth and development as the *vtc4* mutants displayed a short root phenotype while complemented or over-expression lines showed normal or slightly elongated roots at the seedling stage. However, further study is required to understand the role of IMP in root growth and development.

Taken together, our results, in agreement with earlier studies, strongly suggest that seed germination and early seedling growth require the co-ordinate participation of various metabolic pathways including *myo*-inositol, ascorbate, and gluconeogenesis and CaIMP possibly links such pathways to play an important role in improving seed germination and seedling growth, particularly under stressful environments.

## Supplementary data

Supplementary data can be found at *JXB* online.

Supplementary Fig. S1. Diagrammatic representation of the *CaIMP* genomic structure.

Supplementary Fig. S2. Effect of divalent cations on the activity of purified recombinant CaIMP.

Supplementary Fig. S3. Effect of temperature and pH on the activity of purified recombinant CaIMP.

Supplementary Fig. S4. Effect of monovalent cations on the activity of purified recombinant CaIMP.

Supplementary Fig. S5. Sequence of the 5′ upstream regions of the *CaIMP* gene.

Supplementary Fig. S6. Photograph showing the representative germination comparison of *wt*, *vtc4-4*, *vtc4-4:vc*, and *vtc4-4:CaIMP* of *Arabidopsis thaliana* at 10 °C.

Supplementary Table S1. Primers used in this study.

Supplementary Data

## References

[CIT0001] Ahmad F Gaur PM Croser J 2005 Chickpea (*Cicer arietinum* L.) In: Singh R Jauhar P, eds. Genetic resources, chromosome engineering and crop improvement: grain legumes , Vol. 1 Boca Raton, FL, USA: CRC Press, 187–217

[CIT0002] Alexieva V Sergiev I Mapelli S Karanov E 2001 The effect of drought and ultraviolet radiation on growth and stress markers in pea and wheat. Plant, Cell and Environment 24, 1337–1344

[CIT0003] Arrigoni O Calabrese G Gara D Bitonti MB Liso R 1997 Correlation between changes in cell ascorbate and growth of *Lupinus albus* seedlings. Journal of Plant Physiology 150, 302–308

[CIT0004] Atack JR Broughton HB Pollack S 1995 Structure and mechanism of inositol monophosphatase. FEBS Letters 361, 1–7 7890024 10.1016/0014-5793(95)00063-f

[CIT0005] Bailly C 2004 Active oxygen species and antioxidant in seed biology. Seed Science Research 14, 93–107

[CIT0006] Baykov AA Evtushenko OA Avaeva SM 1988 A simple and sensitive colorimetric assay for protein phosphatase activity based on the determination of released Pi by an improved malachite green procedure. Analytical Biochemistry 171, 266–270 3044186 10.1016/0003-2697(88)90484-8

[CIT0007] Boominathan P Shukla R Kumar A Manna D Negi D Verma PK Chattopadhyay D 2004 Long term transcript accumulation during the development of dehydration adaptation in *Cicer arietinum* . Plant Physiology 135, 1608–1620 15247380 10.1104/pp.104.043141 PMC519075

[CIT0008] Bradford MM 1976 A rapid and sensitive method for the quantitation of microgram quantities of protein utilizing the principle of protein–dye binding. Analytical Biochemistry 72, 246–254 10.1016/0003-2697(76)90527-3 942051

[CIT0009] Chagas RM Silveira JAG Rafael V Ribeiro RV Vitorello VA Carrer H 2008 Photochemical damage and comparative performance of superoxide dismutase and ascorbate peroxidase in sugarcane leaves exposed to paraquat-induced oxidative stress. Pesticide Biochemistry and Physiology 90, 181–188

[CIT0010] Chaouch S Noctor G 2010 *myo-*inositol abolishes salicylic acid-dependent cell death and pathogen defense responses triggered by peroxisomal hydrogen peroxide. New Phytologist 188, 711–718 20807338 10.1111/j.1469-8137.2010.03453.x

[CIT0011] Clerkx EJM Blankestijn-DeVries H Ruys GJ Groot SPC Koornneef M 2004 Genetic differences in seed longevity of various *Arabidopsis* mutants. Physiolgia Plantarum 121, 448–461

[CIT0012] Clough SJ Bent AF 1998 Floral dip: a simplified method for *Agrobacterium*-mediated transformation of *Arabidopsis thaliana* . The Plant Journal 16, 735–743 10069079 10.1046/j.1365-313x.1998.00343.x

[CIT0013] Conklin PL 2001 Recent advances in the role and biosynthesis of ascorbic acid in plants. Plant, Cell and Environment 24, 383–394

[CIT0014] Dat J Vandenabeele S Vranová E Montagu M Inzé D Van Breusegem F 2000 Dual action of the active oxygen species during plant stress responses. Cellular and Molecular Life Sciences 57, 779–795 10892343 10.1007/s000180050041 PMC11147059

[CIT0015] Diehl RE Whiting P Potter J Gee N Ragan CI Linemeyer D Schoepfer R Bennett C Dixon AF 1990 Cloning and expression of bovine brain inositol monophosphatase. Journal of Biological Chemistry 256, 5946–5949 1690719

[CIT0016] Donahue JL Alford SR Torabinejad AJ 2010 The *Arabidopsis thaliana myo*-inositol 1-phosphate synthase1 gene is required for *myo*-inositol synthesis and suppression of cell death. The Plant Cell 22, 999–903 10.1105/tpc.109.071779 PMC2861443 20215587

[CIT0017] Downes CP Gray A Lucocq JM 2005 Probing phosphoinositide functions in signaling and membrane trafficking. Trends in Cell Biology 15, 259–268 15866030 10.1016/j.tcb.2005.03.008

[CIT0018] Fu J Peterson K Guttieri M Souza E Raboy V 2008 Barley (*Hordeum vulgare* L.) inositol monophosphatase: gene structure and enzyme characteristics. Plant Molecular Biology 67, 629–642 18493722 10.1007/s11103-008-9343-3

[CIT0019] Gillaspy GE Keddie JS Oda K Gruissem W 1995 Plant inositol monophosphatase is a lithium-sensitive enzyme encoded by a multigene family. The Plant Cell 7, 2175–2185 8718627 10.1105/tpc.7.12.2175 PMC161071

[CIT0020] Graham IA 2008 Seed storage oil mobilization. Annual Reviews of Plant Biology 59, 115–142 10.1146/annurev.arplant.59.032607.092938 18444898

[CIT0021] Gross W Boss WF 1993 Inositol phospholipids and signal transduction. In: Verma DPS, ed. Control of plant gene expression. Boca Raton, FL, USA: CRC Press, 17–32

[CIT0022] Harb A Krishnan A Amvavaram MMR Pereira A 2010 Molecular and physiological analysis of drought stress in *Arabidopsis* reveals early responses leading to acclimation in plant growth. Plant Physiology 154, 1254–1271 20807999 10.1104/pp.110.161752 PMC2971604

[CIT0023] Heath RL Packer L 1968 Photoperoxidation in isolated chloroplast. I. Kinetics and stiochiometry of fatty acid peroxidation. Archives of Biochemistry and Biophysics 125, 189–198 5655425 10.1016/0003-9861(68)90654-1

[CIT0024] Higo K Ugawa Y Iwamoto M Korenaga T 1999 Plant *cis* acting regulatory DNA elements (PLACE) database. Nucleic Acids Research 27, 297–300 9847208 10.1093/nar/27.1.297 PMC148163

[CIT0025] Holmberg N Bülow L 1998 Improving stress tolerance in plants by gene transfer. Trends in Plant Science 3, 61–66

[CIT0026] Ishitani M Majumder AL Bornhouser A Michalowski CB Jensen RG Bohnert HJ 1996 Coordinate transcriptional induction of *myo*-inositol metabolism during environmental stress. The Plant Journal 9, 537–548 8624516 10.1046/j.1365-313x.1996.09040537.x

[CIT0027] Islas-Flores I Villanueva MA 2007 Inositol-1 (or 4)-monophosphatase from *Glycine max* embryo axes is a phosphatase with broad substrate specificity that include phytate dephosphorylation. Biochimica et Biophysica Acta 1770, 543–550 17241743 10.1016/j.bbagen.2006.12.001

[CIT0028] Jiang M Zhang J 2002 Water-stress-induced abscisic acid accumulation triggers the increased generation of reactive oxygen species and up-regulates the activities of antioxidants enzymes in maize leaves. Journal of Experimental Botany 53, 2401–2410 12432032 10.1093/jxb/erf090

[CIT0029] Kampfenkel K Montagu MV Inzé D 1995 Extraction and determination of ascorbate and dehydroascorbate from plant tissue. Analytical Biochemistry 225, 165–167 7778771 10.1006/abio.1995.1127

[CIT0030] Kaur H Shukla RK Yadav G Chattopadhyay D Majee M 2008 Two divergent genes encoding l-*myo*-inositol 1-phosphate synthase 1 (*CaMIPS1*) and 2 (*CaMIPS2*) are differentially expressed in chickpea. Plant, Cell and Environment 31, 1701–1716 10.1111/j.1365-3040.2008.01877.x 18721262

[CIT0031] Kaur H Verma P Petla BP Rao V Saxena SC Majee M 2013 Ectopic expression of ABA-inducible dehydration-responsive chickpea l-*myo*-inositol 1-phosphate synthase 2 (*CaMIPS2*) in *Arabidopsis* enhances tolerance to salinity and dehydration stress. Planta 237, 327–335 10.1007/s00425-012-1781-0 23065054

[CIT0032] Laemmli UK 1970 Cleavage of structural proteins during the assembly of the head of bacteriophage T4. Nature 227, 680–685 5432063 10.1038/227680a0

[CIT0033] Laing WA Bulley S Wright M Cooney J Jensen D Barraclough D MacRae E 2004 A highly specific l-galactose-1-phosphate phosphatase on the path to ascorbate biosynthesis. Proceeding of the National Academy of Sciences, USA 101, 16976–16981 10.1073/pnas.0407453101 PMC534719 15550539

[CIT0034] Lescot M Dehais P Thijs G Marchal K Moreau Y Van de Peer Y Rouzé P Rombauts S 2002 PlantCARE, a database of plant *cis* acting regulatory elements and a portal of tools for *in silico* analysis of promoter sequences. Nucleic Acids Research 30, 325–327 11752327 10.1093/nar/30.1.325 PMC99092

[CIT0035] Lichtenthaler HK Wellburn AR 1983 Determination of total carotenoids and chlorophylls A and B of leaf in different solvents. Biochemical Society Transactions 11, 591–592

[CIT0036] Loewus FA 1990 Inositol biosynthesis. In: Morre DJ Boss WF Loewus FA, eds. Inositol metabolism in plants. New York: Wiley-Liss, 13–19

[CIT0037] Loewus FA Loewus MW 1983 *myo-*Inositol: its biosynthesis and metabolism. Annual Review of Plant Physiology 34, 137–161

[CIT0038] Loewus FA Murthy PN 2000 *myo-*Inositol metabolism in plants. Plant Science 150, 1–19

[CIT0039] Majee M Maitra S Dastidar KG Pattnaik S Chatterjee A Hait NC Das KP Majumder AL 2004 A novel salt-tolerant l-*myo*-inositol 1-phosphate synthase from *Porteresia coarctata* (Roxb.) Tateoka, a halophytic wild rice: molecular cloning, bacterial over-expression, characterization, and functional introgression into tobacco conferring salt-tolerant phenotype. Journal of Biological Chemistry 279, 28539–28552 15016817 10.1074/jbc.M310138200

[CIT0040] Meng PH Raynaud C Tcherkez G 2009 Crosstalks between *myo*-inositol metabolism, programmed cell death and basal immunity in *Arabidopsis* . PLoS One 4, e7364 19812700 10.1371/journal.pone.0007364 PMC2754662

[CIT0041] Neuwald AF Krishnan BR Brikun I Kulakauskas S Suziedelis K Tomcsanyi K Leyh TS Berg DE 1992 cysQ, a gene needed for cysteine synthesis in *Escherichia coli* K-12 only during aerobic growth. Journal of Bacteriology 174, 415–425 1729235 10.1128/jb.174.2.415-425.1992 PMC205732

[CIT0042] Nunes ACS Vianna GR Cuneo F Amaya-Farfa’n J Capdeville G Rech EL Aragao FJL 2006 RNAi mediated silencing of the *myo-*inositol 1-phosphate synthase gene (*GmMIPS1*) in transgenic soybean inhibited seed development and reduced phytate content. Planta 224, 125–132 16395584 10.1007/s00425-005-0201-0

[CIT0043] Padh H 1990 Cellular functions of ascorbic acid. Biochemistry and Cell Biology 68, 1166–1173 2268411 10.1139/o90-173

[CIT0044] Parthasarathy R Parthasarathy L Vadnal R 1997 Brain inositol monophosphatase identified as a galactose 1-phosphatase. Brain Research 778, 99–106 9462881 10.1016/s0006-8993(97)01042-1

[CIT0045] Patra B Ghosh Dastidar K Maitra S Bhattacharya J Majumder AL 2007 Functional identification of sll1383 from *Synechocystis* sp. PCC 6803 as l-*myo*-inositol 1-phosphate phosphatase (EC 3.1.3.25): molecular cloning, expression and characterization. Planta 225, 1547–1558 17123102 10.1007/s00425-006-0441-7

[CIT0046] Petersen LN Marineo S Mandalà S Davids F Sewell BT Sewell BT Ingle RA 2010 The missing link in plant histidine biosynthesis: *Arabidopsis myo-*inositol monophosphatase-like 2 encodes a functional histidinol-phosphatase. Plant Physiology 152, 1186–1196 20023146 10.1104/pp.109.150805 PMC2832243

[CIT0047] Prochazkova D Sairam RK Srivastava GC Singh DV 2001 Oxidative stress and antioxidant activity as the basis of senescence in maize leaves. Plant Science 161, 765–771

[CIT0048] Quintero FJ Garciadeblás B Rodríguez-Navarro A 1996 The SAL1 gene of *Arabidopsis*, encoding an enzyme with 3′(2′),5′-*bis*phosphate nucleotidase and inositol polyphosphate 1-phosphatase activities, increases salt tolerance in yeast. The Plant Cell 8, 529–537 8721754 10.1105/tpc.8.3.529 PMC161118

[CIT0049] Raboy V 2003 *myo*-Inositol-1,2,3,4,5,6-hexakisphosphate. Phytochemistry 64, 1033–1043 14568069 10.1016/s0031-9422(03)00446-1

[CIT0050] Raboy V 2001 Seeds for a better future: ‘low phytate’ grains help to overcome malnutrition and reduce pollution. Trends in Plant Science 6, 458–462 11590064 10.1016/s1360-1385(01)02104-5

[CIT0051] RayChaudhuri A Majumder AL 1996 Salinity-induced enhancement of 1-*myo*-inositol 1-phosphate synthase in rice (*Oryza sativa* L.). Plant, Cell and Environment 19, 1437–1442

[CIT0052] Sambrook J Russell DW 2001 Molecular cloning: a laboratory manual , 3rd edn, Vol. 3: *Expression of cloned genes in* Escherichia coli. Cold Spring Harbor, NY, USA: Cold Spring Harbor Laboratory Press, 15.23–15.24

[CIT0053] Saxena SC Joshi PK Grimm B Arora S 2011 Alleviation of ultraviolet-C-induced oxidative damage through overexpression of cytosolic ascorbate peroxidase. Biologia 66, 1052–1059

[CIT0054] Schonhaf I Kläring HP Krumbein A Claußben W Schreiner M 2007 Effect of temperature increase under low radiation conditions on phytochemicals and ascorbic acid in greenhouse-grown broccoli. Agriculture, Ecosystem and Environment 119, 103–111

[CIT0055] Singh KB Ocampo B Robertson LD 1998 Diversity for abiotic and biotic stress resistance in the wild annual *Cicer* species. Genetic Resources and Crop Evolution 45, 9–17

[CIT0056] Smirnoff N 1995 Antioxidant system and plant response to the environment. In: Smirnoff N, ed. Environment and plant metabolism: flexibility and acclimation. Oxford, UK: Bios Scientific Publishers, 217–243

[CIT0057] Stasolla C Yeung EC 2001 Ascorbic acid metabolism during white spruce somatic embryogenesis. Physiologia Plantarum 111, 196–205

[CIT0058] Stec B Yang H Johnson KA Chen L Roberts MF 2000 MJ0109 is an enzyme that is both an inositol monophosphatase and the ‘missing’ archaeal fructose-1,6-*bi*sphosphatase. Nature Structural Biology 7, 1046–1050 10.1038/80968 11062561

[CIT0059] Styer JC Keddie J Spence J Gillaspy GE 2004 Genomic organization and regulation of the *LeIMP-1* and *LeIMP-2* genes encoding *myo*-inositol monophosphatase in tomato. Gene 326, 35–41 14729261 10.1016/j.gene.2003.09.048

[CIT0060] Suzuki M Tanaka K Kuwano M Yoshida KT 2007 Expression pattern of inositol phosphate-related enzymes in rice (*Oryza sativa* L.): implications for the phytic acid biosynthetic pathways. Gene 405, 55–64 17961936 10.1016/j.gene.2007.09.006

[CIT0061] Torabinejad J Donahue JL Gunesekera BN Allen-Daniels MJ Gillaspy GE 2009 VTC4 is a bifunctional enzyme that affects *myo*-inositol and ascorbate biosynthesis in plants. Plant Physiology 150, 951–961 19339506 10.1104/pp.108.135129 PMC2689953

[CIT0062] Verma P Kaur H Petla BP Rao V Saxena SC Majee M 2013 Protein l-isoaspartyl methyltransferase 2 is differentially expressed in chickpea and enhances seed vigor and longevity by reducing abnormal isoaspartyl accumulation predominantly in seed nuclear proteins. Plant Physiology 161, 1141–1157 23284083 10.1104/pp.112.206243 PMC3585586

[CIT0063] Vertucci CW Farrant JM 1995 Acquisition and loss of desiccation tolerance. In: Kigel J Galilli G, eds. Seed development and germination. New York, NY: Marcel Dekker, 237–271

