# Supplementary material for: Differentially expressed myo-inositol monophosphatase gene (CaIMP) in chickpea (Cicer arietinum L.) encodes a lithium-sensitive phosphatase enzyme with broad substrate specificity and improves seed germination and seedling growth under abiotic stresses
Source: J Exp Bot. 2013 Oct 11;64(18):5623–39. doi: 10.1093/jxb/ert336 (PMC3871819; doi:10.1093/jxb/ert336)
Supplement: Supplementary Data [file supp_64_18_5623__index.html]

Differentially expressed myo-inositol monophosphatase gene (CaIMP) in chickpea (Cicer arietinum L.) encodes a lithium-sensitive phosphatase enzyme with broad substrate specificity and improves seed germination and seedling growth under abiotic stresses — Differentially expressed myo-inositol monophosphatase gene (CaIMP) in chickpea (Cicer arietinum L.) encodes a lithium-sensitive phosphatase enzyme with broad substrate specificity and improves seed germination and seedling growth under abiotic stresses — Supplementary Data 

# Differentially expressed *myo*-inositol monophosphatase gene (*CaIMP*) in chickpea (*Cicer arietinum* L.) encodes a lithium-sensitive phosphatase enzyme with broad substrate specificity and improves seed germination and seedling growth under abiotic stresses

## Supplementary Data

Data files

**Files in this Data Supplement:**

- Supplementary Data - Supplementary Data
