# Supplementary material for: Differentially expressed myo-inositol monophosphatase gene (CaIMP) in chickpea (Cicer arietinum L.) encodes a lithium-sensitive phosphatase enzyme with broad substrate specificity and improves seed germination and seedling growth under abiotic stresses
Source: J Exp Bot. 2013 Oct 11;64(18):5623–39. doi: 10.1093/jxb/ert336 (PMC3871819; doi:10.1093/jxb/ert336)
Supplement: Supplementary Data [file supp_ert336_jexbot105965_file001.pdf]

Title: Differentially Expressed *myo*-Inositol Monophosphatase Gene (*CaIMP*) in Chickpea (*Cicer arietinum* L.) Encodes A Lithium Sensitive Phosphatase Enzyme with Broad Substrate Specificity and Improves Seed Germination and Seedling Growth under Abiotic Stresses

Authors:

Name: Saurabh C Saxena, Prafull Salvi, Harmeet Kaur, Pooja Verma, Bhanu Prakash Petla, Venkateswara Rao, Nitin Kamble and Manoj Majee

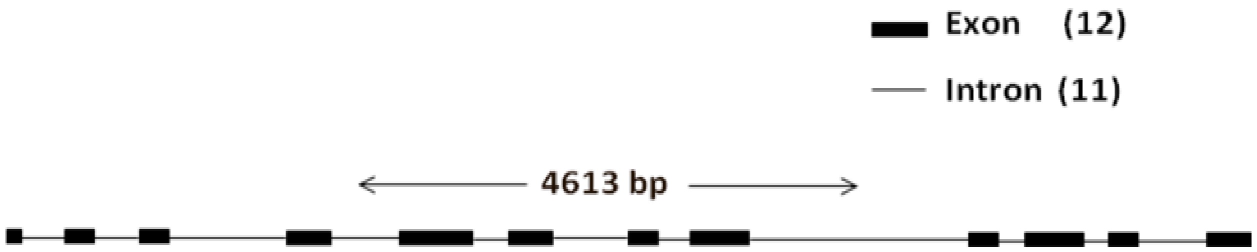

Supplemental Figure S1: Diagrammatic representation of *CaIMP* genomic structure. The boxes represent exons and the lines between them represent introns.

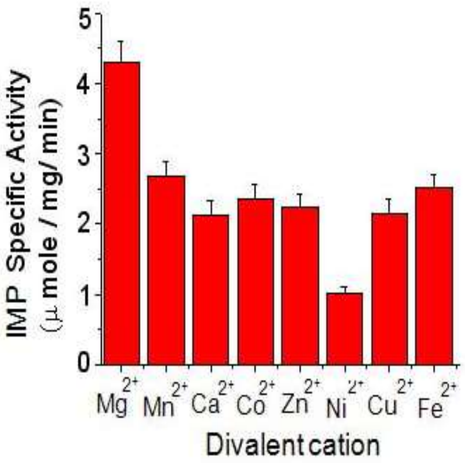

Supplemental Figure S2. Effect of divalent cation on the activity of purified recombinant CaIMP using inositol 1-phosphate as substrate. IMP activity was determined in presence of 5mM of each cation. In each case, 10μg purified protein was used. Specific activity was calculated μmole Pi released per mg of protein per minute. In each case values are mean ± SE of three replicates.

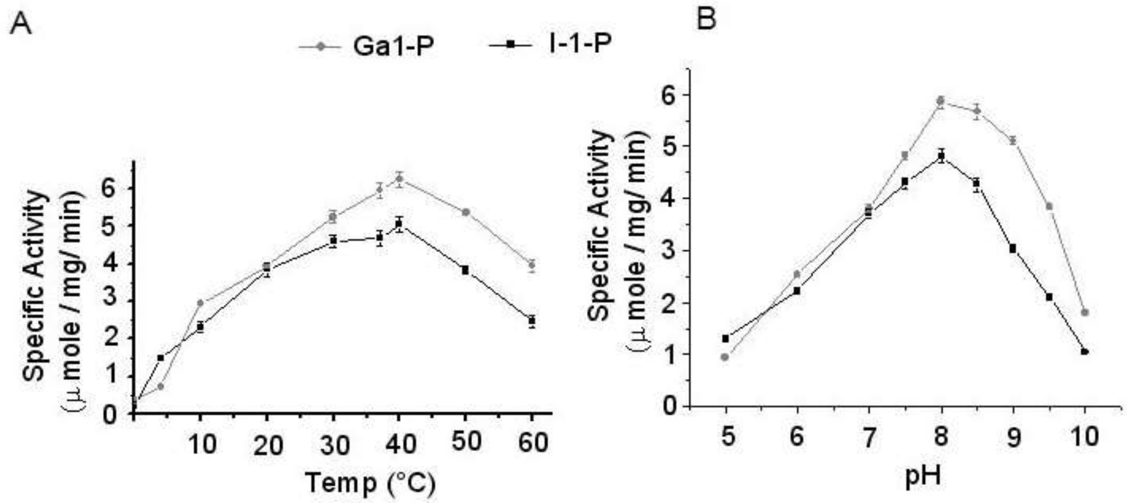

Supplemental Figure S3. (**A)** Effect of temperature on the activity of purified recombinant CaIMP. The specific activity was measured at different temperatures ranging from 0 to 60°C. In each case values are mean ± SE of three replicates.

(B) Effect of pH on the activity of purified recombinant CaIMP. The specific activity was measured at 37°C at a pH range of 5 to 10. In each case, 10μg purified protein was used and values are mean ± SE of three replicates.

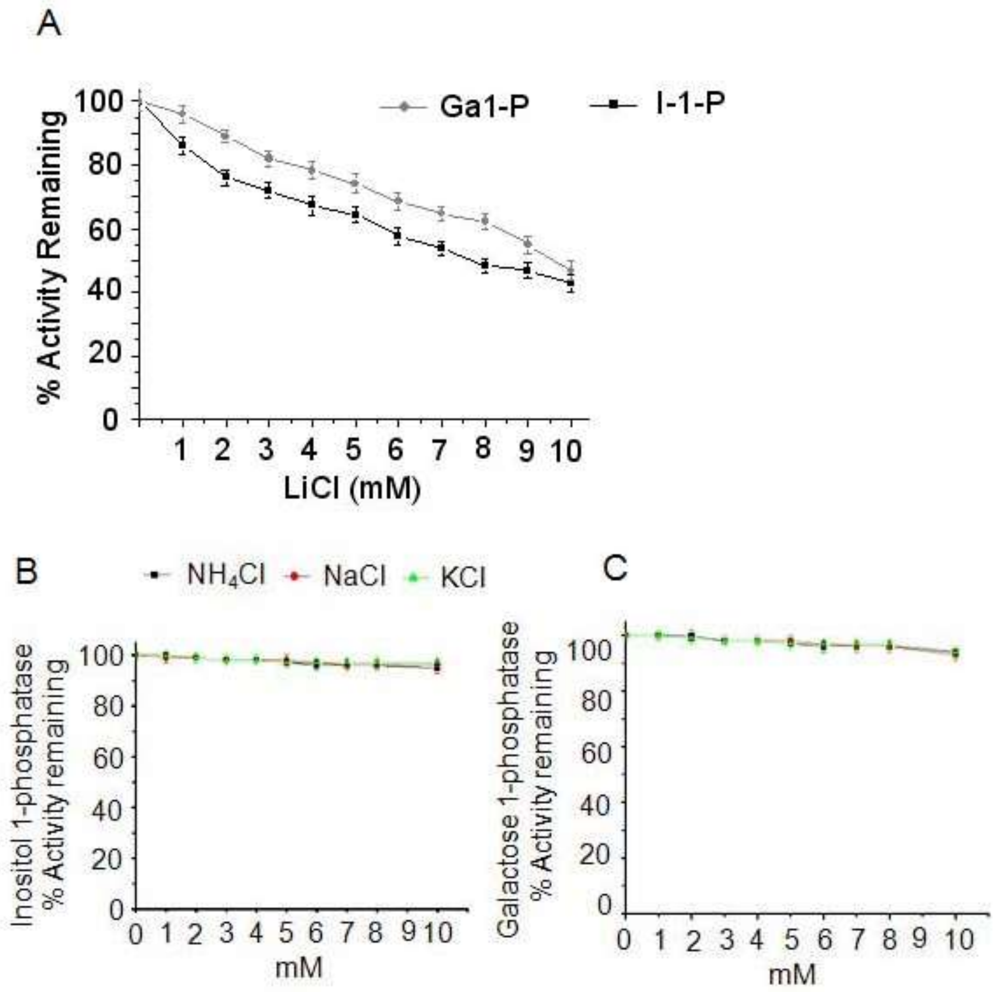

Supplemental Figure S4. (A) Effect of Li^+ on the activity of purified recombinant CaIMP. activity was assessed with increasing concentration of lithium chloride (LiCl) solution.

(B-C) Effect of monovalent cation (NH_4^+,Na^+, K^+) on the activity of purified recombinant CaIMP. **(B)** IMP activity and **(C)** galactose 1-phosphatase activity was determined in presence of increasing concentration (0-10mM) of salt. In each case, 10μg purified protein was used. In each case values are mean ± SE of three replicates.

CCTCTTTTGTATAACACTCTTATATTGACTTTAATGCTTAGATGATCAACATATCTCTCTCAAA

Auxin RE

ACATTAGAGAATCCAAATACCCGTAAAACTTTGTATACAATTTATTAAAGAAGAATTCAATGTA

DOF binding

TCTTATAAATGTAATAATAATTCTTTTAAAAAAAATAATTGTAATAATAATAACAATTTAGTCA

Gibberellin RE

ATAATAATAACAAACGGTATGTCATCTTCTTTCATATTTTGATCATTTGGTAATGTAAATGTTA

Gibberellin RE **TCA1MOTIF (Salicylic acid RE)**

TTCTCATGCATTGAATTTCACGGTATCTTCTGAATGAATTTATGAGCTCTCTTGTAGCAACCGA

RY repeat motif

AGTATCTTAAACGAATTTGGACCATTTTAATTTAAAATATTTCTCAATCTCTGTCAATTATTTT

aATTTTAATTTTTTTTATTAGATCAAAAGTTTATATTGTATAAATTACGCATTTAAAATTTTAT

Cytokinin RE **DOF binding**

ATAAATTTAAAATCATTTAATATGTTATTAAAATCCATGAATATTAACAATATTTAATAAAACT

Gibberellin RE

ACATACCACATTAATTTTGATGTGTCACCGACATATCAAATGATTTTAAATTTATACAAAATTT

Light RE **DRE/CRT (Low Temperature/ Drought RE)**

TATATGAATAATTTATACGATATAAACTTTCGATTAAACGAAATTTGTTAAAAACTTTATATGC

Auxin RE

ATTATCTTTTATGTTTGTAAATATTTATATTGATAATCAAAATTTAAAACTAGCAAAATTAAAA

CTCAATTTTTTTAATAAACTCATGTAATTCTTAAAGAGTTTTGATTGCAATAACCCTAAGAATA

DOF binding

TAAAACCTCAATCAATTTTTAGAAAAATAATTAAATCAGAGAGAAGTCAAGTGAGATACACAGT

Salt/pathogen RE **MYCCONSENSUSAT (Drought RE)**

AGAATTTTTTAAATTAAATATGGAATTCTCTTAATTCAATTCATGAAATTACAAAAATATTATT

ATTTTGTTCTTTATATAATCGATAATAAAATTCATAGTAAAATTTTAAAAATTTATAATTAAAA

Heat RE

CTAATTGAAACGATGTTATGACTAAAAATAATTAAAAGACTCTTAAATTTAATTTATCTATCTA

WBOXHVISO1 (Sugar RE) **DOF binding**

ATTAATAAATAATTTATACACTATCAAATCAAATCGCAGTGATTAGATCATTAATTATATTTAG

Light RE **Light RE**

CTTTTAATATAATAACTTATAAAGTCACGCTCATAGACCTGATCGACCCTGATTTATTGATAAT

IBOXCORE (Light RE) **IBOXCORE (Light RE)** **IBOXCORE (Light RE)**

ATCTAACTTTTTACCGTGAGAATGAATAAAAATTAAAGTATTTCACAATTAGAGTATAAAGaAA

IBOXCORE (Light RE) **DOF binding** **DOF binding**

AAAAAAAAAAAAGAATATAGTGATGCGATGGTATATTCTAGTACTATTTGATTCTAGCTAAGAG

DOF binding **P1BS (Phosphate Starvation RE)**

CGATCATCTACACATAAATTAAAACTAAGAGCACATACGGTTTTGTGTATGGTGAGTGGTTAGT

GCGTTACGCGAAAGGAAGGATCAATAACAAACACACAGCCTCTTCCTTCCATATCATCTCTCTC

DOF binding **Gibberellin RE/ Endosperm expression**

TCTCTCTCTCTCTCTCTCTCTCTCTCTCTCTCTCTCTCTCTCTCTCTCTACAATTCCACATCAA

AATG

Supplemental Figure S5. Sequence of the 5' upstream regions of *CaIMP* gene. Nucleotide sequences representing major potential *cis*- regulatory elements are mentioned and underlined. Translational start codon (ATG) is highlighted.

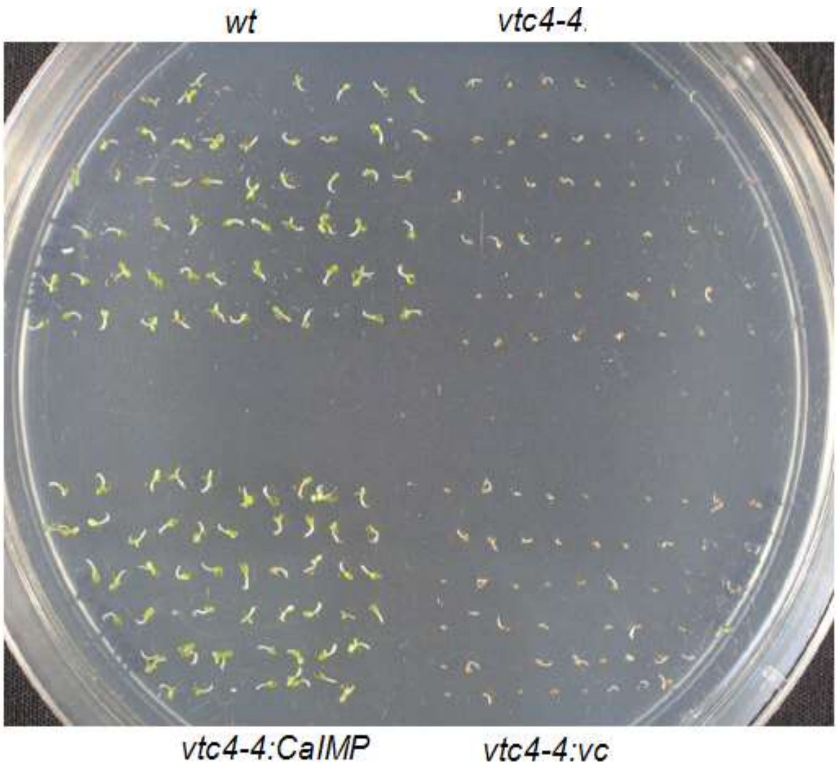

Supplemental Figure S6. Photograph showing the representative germination comparison of *wt*, *vtc4-4*, *vtc4-4: vc* and *vtc4-4:CaIMP* of *Arabidopsis thaliana* at 10°C

Primer No.	Primer sequence (5'-3')	Purpose
CaIMP1 F	GGMACIACTAAYTTTGTACATGG	*CaIMP* 3' RACE
CaIMP2 F	TGGIGCTGTCATTGTTAGRGAAGC	
CaIMP3R	CATTCCGTTTGGCGCAGAGCATCAAC	*CaIMP* 5' RACE
CaIMP4R	ATTCAAAAATGCACCTTGTCCACG	
CaIMP5 F	GGAAGGATCAATAACAAACACACAG	To clone full length *CaIMP* genomic sequence
CaIMP6 R	CCGACTAGTATCGGAATCAATAG	
CaIMP7F	GGCATATGGTTGACAATGATTCACACTC	To clone full length CDS of *CaIMP* in bacterial expression vector
CaIMP8R	CTCGAGTTCCGTTTGGCGCAGAGCATC	
CaIMP9F	AGCGTGTAGCTGCTTCAAACC	Real time PCR in chickpea for *CaIMP1*
CaIMP10R	GTTTGGCGCAGAGCATCA	
MM 160 F	GCCCGCGACGTTGTGA	Real time PCR for chickpea 18S (Endogenous control)
MM 161 R	CCTTGTTACGACTTCTCCTTCCTCTA	
RTMIPS1F	TGAGAATATGTTTGCTGCTGATGTT	Real Time PCR in chickpea for *CaMIPS1*
RTMIPS1R	AAAGCACCAACGAGCAAAGAC	
RTMIPS2F	TGGATTGGCTCCTGAGAACAA	Real Time PCR in chickpea for *CaMIPS2*
RTMIPS2R	CCCAAATTCCTAACTGAATCGTTAC	
CaIMP11F	GGAGCTCATGGTTGACAATGATTCACACTC	For preparing Hygromycin construct of *CaIMP* (mutant complementation)
CaIMP12 R	CTCTAGATCATTCCGTTTGGCGCAGAGCATC	
CaIMP13 F	GAAGCTTATGGTTGACAATGATTCACACTC	To clone full length CDS of *CaIMP* in plant expression vector for 35S overexpression lines
CaIMP12 R	CTCTAGATCATTCCGTTTGGCGCAGAGCATC	
CaIMP14 R	TGATGTGGAATTGTAGAGAGAGAGAG	For *CaIMP* genome walking
CaIMP15 R	AGAGAGAGATGATATGGAAGGAAGAG	
CaIMP16F	GGCCCATCCATGTATGTTCTTGACC	For cloning *CaIMP* promoter (1.5 Kb)
CaIMP14R	TGATGTGGAATTGTAGAGAGAGAGAG	

Supplemental Table 1. Primers used in this study
